# DNA Assembly of Modular Components into a Rotary Nanodevice

**DOI:** 10.1021/acsnano.1c10160

**Published:** 2022-03-14

**Authors:** Andreas Peil, Ling Xin, Steffen Both, Luyao Shen, Yonggang Ke, Thomas Weiss, Pengfei Zhan, Na Liu

**Affiliations:** † Second Physics Institute, University of Stuttgart, Pfaffenwaldring 57, 70569 Stuttgart, Germany; ‡ Max Planck Institute for Solid State Research, Heisenbergstrasse 1, 70569 Stuttgart, Germany; § Fourth Physics Institute, University of Stuttgart, Pfaffenwaldring 57, 70569 Stuttgart, Germany; ∥ Wallace L. Coulter Department of Biomedical Engineering, Emory University and Georgia Institute of Technology, Atlanta, Georgia 30322 United States; ⊥ Institute of Physics, University of Graz, and NAWI Graz, Universitätsplatz 5, 8010 Graz, Austria

**Keywords:** self-assembly, DNA origami, nanoparticles, nanoscale rotary motion, fluorescence spectroscopy

## Abstract

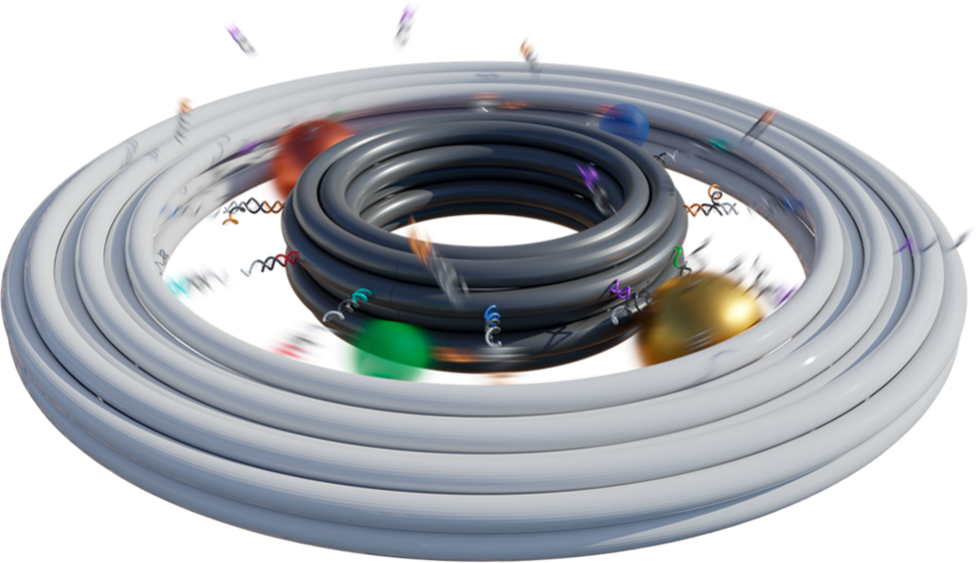

The bacterial flagellar motor is a rotary machine composed of functional modular components, which can perform bidirectional rotations to control the migration behavior of the bacterial cell. It resembles a two-cogwheel gear system, which consists of small and large cogwheels with cogs at the edges to regulate rotations. Such gearset models provide elegant blueprints to design and build artificial nanomachinery with desired functionalities. In this work, we demonstrate DNA assembly of a structurally well-defined nanodevice, which can carry out programmable rotations powered by DNA fuels. Our rotary nanodevice consists of three modular components, small origami ring, large origami ring, and gold nanoparticles (AuNPs). They mimic the sun gear, ring gear, and planet gears in a planetary gearset accordingly. These modular components are self-assembled in a compact manner, such that they can work cooperatively to impart bidirectional rotations. The rotary dynamics is optically recorded using fluorescence spectroscopy in real time, given the sensitive distance-dependent interactions between the tethered fluorophores and AuNPs on the rings. The experimental results are well supported by the theoretical calculations.

## Introduction

Cells contain a large multitude of molecular machines, which support and sustain vital biological functions.^1^ One class of the molecular machinery is the rotary motors, for instance, F_1_F_o_-adenosine triphosphate synthases^2−6^ and bacterial flagellar motors.^7−11^ The bacterial flagellum is a motility organelle that rotates and acts as a propeller in many bacteria. It self-assembles from intricate modular components, including rotor-stator that powers flagellar rotation, chemotaxis apparatus that mediates changes in direction, among others.^12,13^ Recent studies have proposed and validated the rotation mechanism that the flagellar motor resembles a two-cogwheel gear system, composed of small and large cogwheels with cogs at the edges to switch their relative positions.^11,14,15^ Such nature’s machine models provide inspiring insights into creation of synthetic molecular devices, which exhibit biomimetic functions and meanwhile may go beyond limitations of natural systems. For instance, integration of nonbiological modular components, that is, nanocrystals, carbon nanotubes, polymersomes, arbitrarily shaped DNA origami structures, *etc*., can substantially enlarge the degrees of freedom to design artificial dynamic systems and small-scale robotics with tailored optical, magnetic, electrical, and many other properties.

Among different approaches for creation of bioinspired nanodevices, the DNA origami technology represents a versatile assembly tool.^16−22^ Single-stranded DNA scaffold molecules are folded by hundreds of shorter DNA strands into target structures with nanoscale addressability, programmability, and spatiotemporal accuracy. Over the past decade, a variety of DNA-based dynamic systems have been implemented, including DNA walkers,^23−28^ sliders,^29−33^ rotary devices,^34−41^ assembly lines,^42^ and cargo sorters,^43^ among others.

In this work, we demonstrate a DNA-assembled planetary gearset nanodevice, containing multiple rotary modules that are compactly linked together. Compared to previously demonstrated rotary nanodevices, our system exhibits increased structural complexity and cooperativity among the modular components. In our nanodevice, the small origami ring, large origami ring, and gold nanoparticles (AuNPs) resemble the sun gear, ring gear, and planet gears in a planetary gearset accordingly. These rotary modules are tightly orchestrated to yield programmable rotations powered by DNA fuels. We optically track the rotation dynamics of the rotary nanodevices using fluorescence spectroscopy in real time. The experimental results are supported by theoretical calculations.

## Results and Discussion

### Assembly of the Rotary Nanodevice

**Figure 1:**
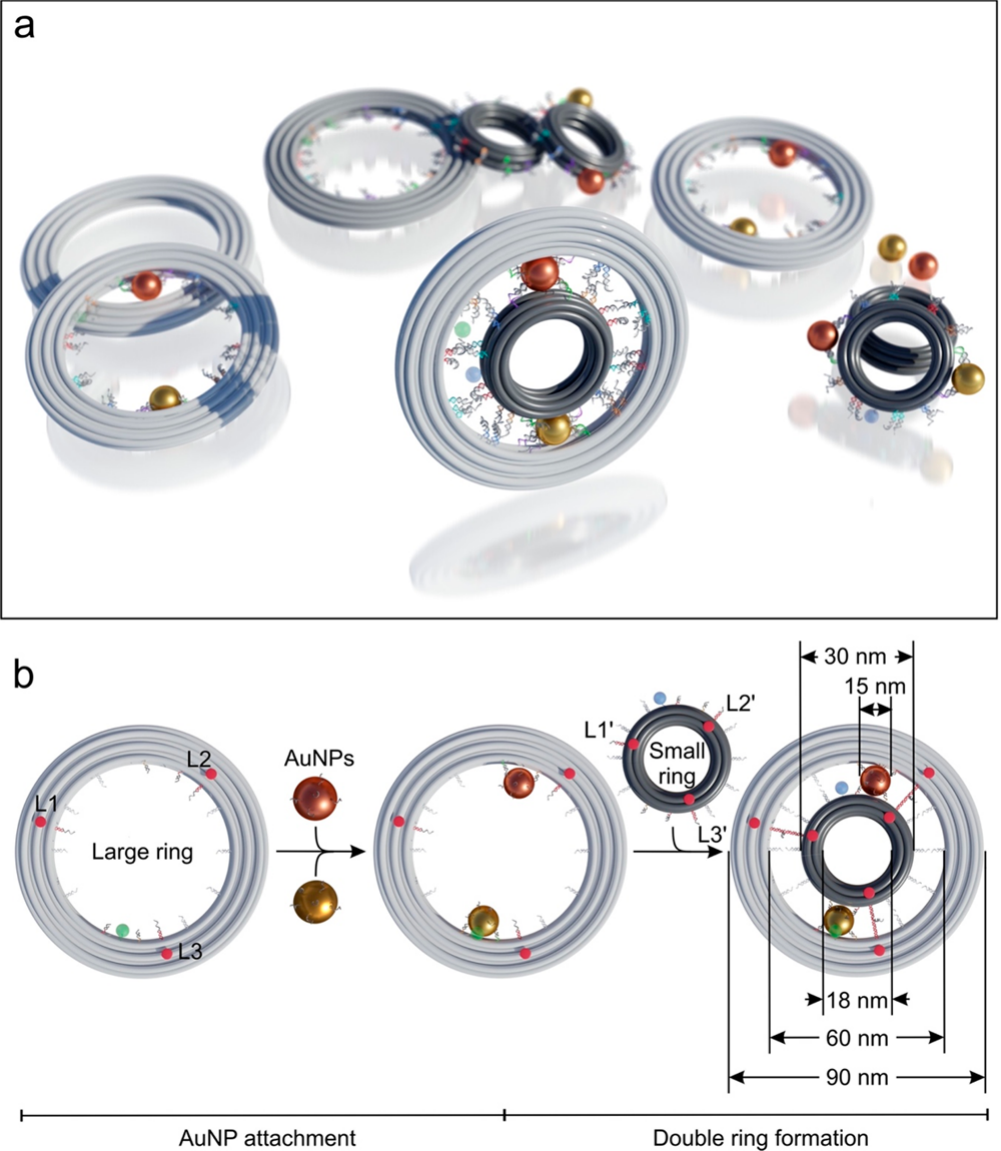
(a) Schematic of the DNA-assembled rotary structures and their modular components, which consist of small (dark gray) and large (light gray) DNA origami rings as well as AuNPs (brown and golden) and fluorophores (blue and green). (b) Planetary gearset is formed by connecting two origami rings through locking of the three locking strands (L1-L1′, L2-L2′, and L3-L3′) and cross-linking of the two AuNPs in between. The small and large rings serve as sun gear and ring gear, respectively, and the AuNPs work as planet gears. Two fluorophores ATTO550 (blue) and ATTO647N (green) are tethered on the small and large rings, respectively.

Figure 1a shows the schematic of the DNA-assembled rotary structures and their modular components, which include small origami rings (dark gray), large origami rings (light gray), gold nanoparticles (AuNPs, brown, and golden), and fluorophores (green and blue). The origami rings are formed through DNA self-assembly processes^16−22^ (see Supporting Information (SI) Figures S1 and S2 for the DNA origami designs). As depicted in Figure 1b, the 10-helix small ring has an outer diameter of ∼30 nm and the 12-helix large ring has an inner diameter of ∼60 nm. The diameter of the AuNPs is 15 nm. To form a planetary gearset nanosystem, the small and large rings are cross-linked together by AuNPs. The small and large rings serve as sun gear and ring gear, respectively, and the AuNPs work as planet gears. For position clarity, the two AuNPs attached on the rings are differentiated using brown and golden colors, respectively.

**Figure 2:**
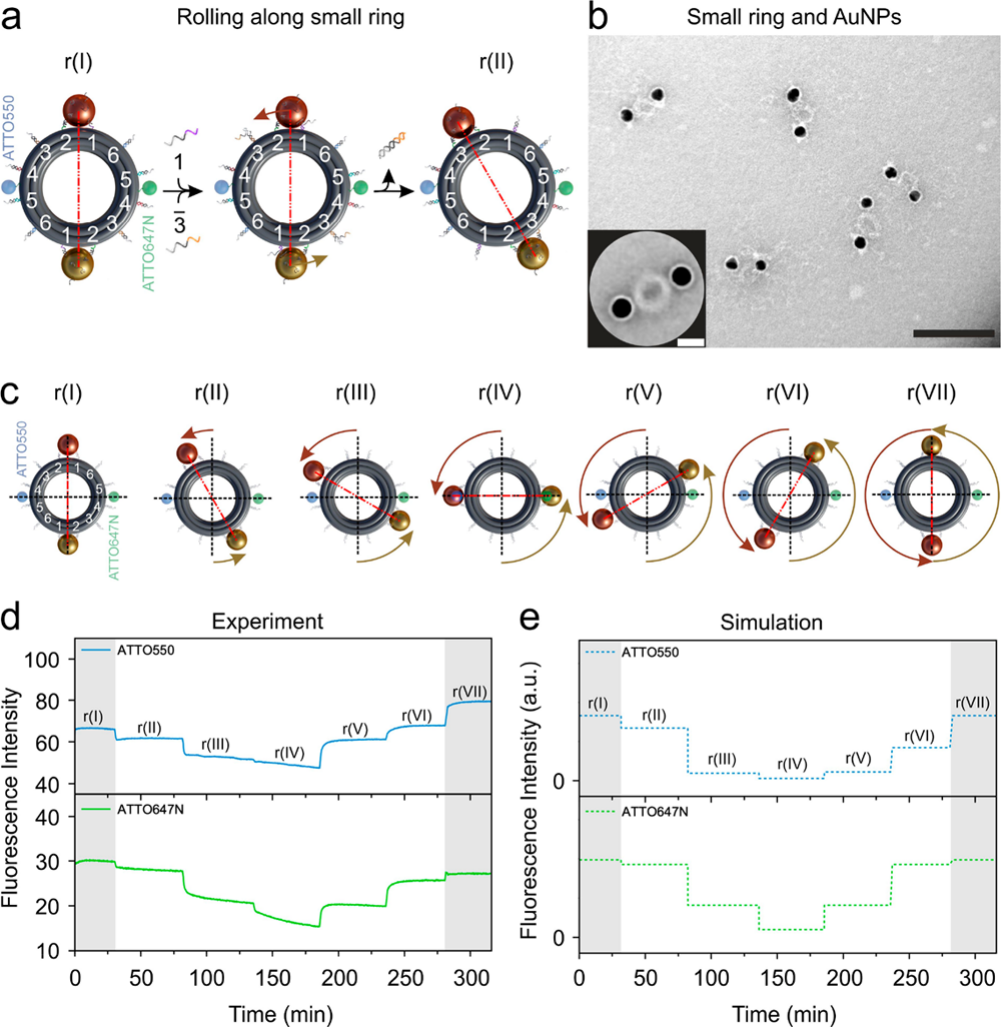
(a) Rolling mechanism of the AuNPs along the small ring through toehold-mediated strand displacement reactions. At “r(I)”, both AuNPs are attached between foothold rows 1 and 2 outside the ring. Both ATTO550 (blue) and ATTO647N (green) are tethered between foothold rows 4 and 5 on the ring. (b) TEM image of the structures. Scale bar, 100 nm. Inset: averaged TEM image. Scale bar, 20 nm. (c) Representative route for the AuNPs rolling along the small ring counterclockwise. “r(I)”–“r(VII)” represent different states. The exact angle change for each step is indicated in SI Figure S4. (d) Fluorescence signals recorded at wavelengths 576 and 664 nm with excitation wavelengths at 554 and 646 nm for ATTO550 and ATTO647N, respectively. (e) Calculated fluorescence results for the different states in (d).

**Figure 3:**
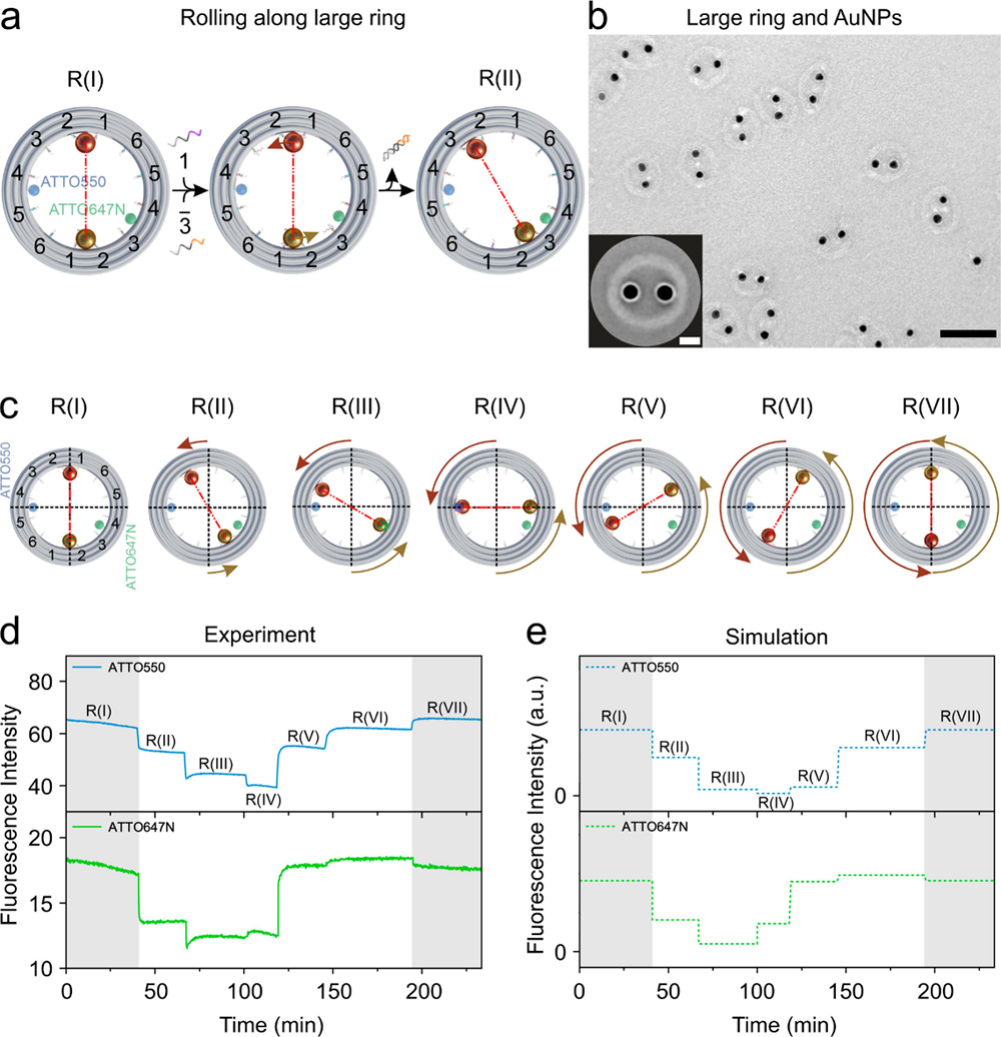
(a) Rolling mechanism of the AuNPs along the large ring through toehold-mediated strand displacement reactions. At “R(I)”, both AuNPs are attached between foothold rows 1 and 2 inside the ring. ATTO550 (blue) is tethered between foothold rows 4 and 5, while ATTO647N (green) is tethered between foothold rows 3 and 4. (b) TEM image of the structures. Scale bar, 100 nm. Inset: averaged TEM image. Scale bar, 20 nm. (c) Representative route for the AuNPs rolling counterclockwise along the large ring. “R(I)”–“R(VII)” represent different states. The exact angle change for each step is indicated in SI Figure S3. (d) Fluorescence signals at different states recorded during the rolling process. (e) Calculated fluorescence results for the different states in (d).

**Figure 4:**
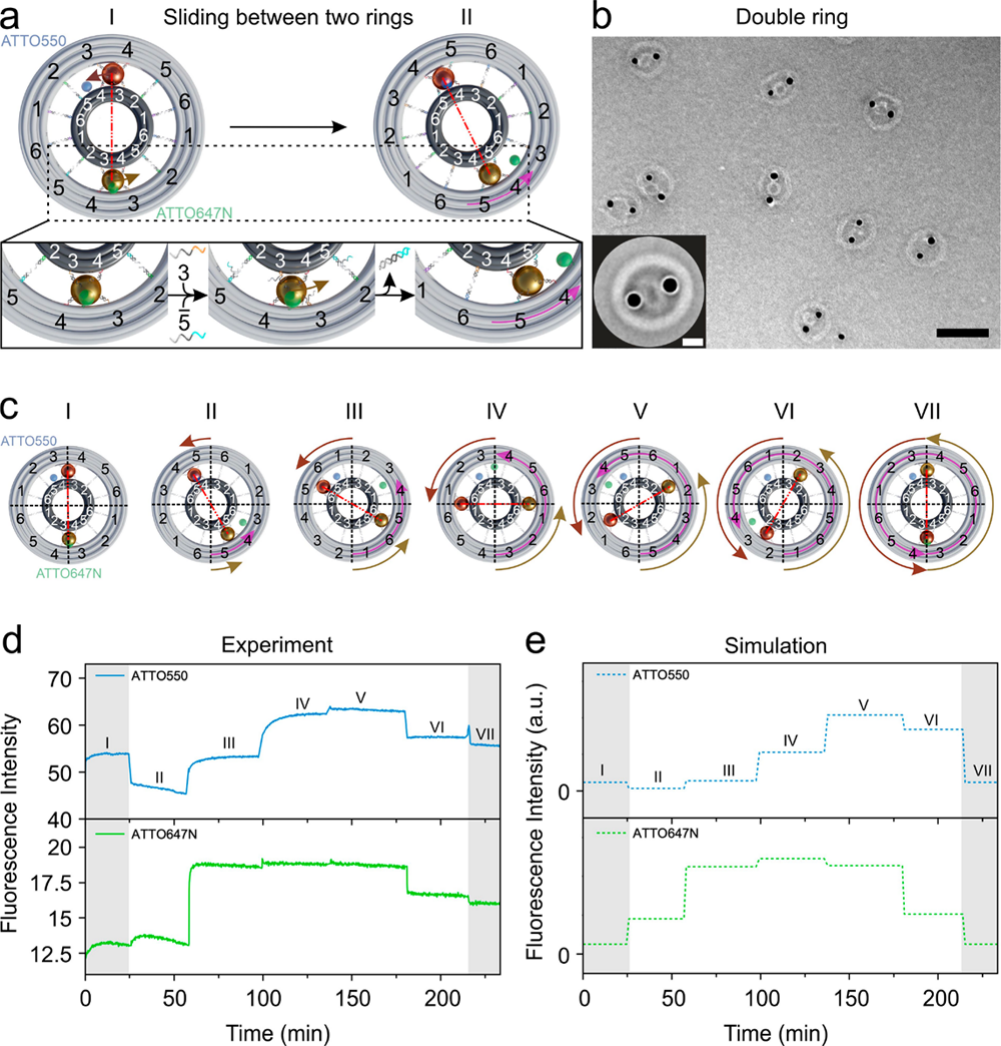
(a) Two AuNPs as planet gears sliding in between the small ring (sun gear) and large ring (ring gear) in a planetary gearset nanodevice. ATTO550 (blue) is tethered on the small ring between foothold rows 4 and 5, while ATTO647N (green) is attached to the large ring between foothold rows 3 and 4. (b) TEM image of the structures. Scale bar, 100 nm. Inset: averaged TEM image. Scale bar, 20 nm. (c) Representative route for the AuNPs sliding in between the two rings. “I”–“VII” correspond to different states. The relative movement between the small and large rings imposed by the AuNPs along opposite directions introduces twice of the angle change compared to that of the AuNPs. This is highlighted by the pink arrow on the large ring at each step. (d) Fluorescence signals recorded at different states during the process. (e) Calculated fluorescence results for the different states in (d).

The large ring is functionalized with 12 rows of foothold strands (see also SI Figures S3 and S5)^31^ as well as three locking strands (L1, L2, L3) along its inner circumference. There are three DNA strands along each foothold row. The locking strands of L1, L2, and L3 are separated by ∼120° around the large ring (see SI Figure S6) and their positions are indicated using red dots in Figure 1b. Two AuNPs fully decorated with DNA (foot strands) are attached to the large ring through DNA hybridization. Each AuNP is bound to two rows of footholds, while the rest of the footholds are deactivated. The specific arrangement of the footholds on the rings and the foot–foothold interactions will be explained later in detail in Figures 2, 3, and 4 successively. The small ring is also functionalized with 12 rows of footholds and three complementary locking strands (L1′, L2′, L3′) along its outer circumference (see SI Figures S4–S6). The small and large rings are subsequently connected to form a planetary gearset. The correct orientations and relative positions of the two rings are enforced by the locking between L1-L1′, L2-L2′, and L3-L3′ as well as the cross-linking of the two AuNPs in between. Specifically, each AuNP is bound to four rows of footholds, two from the small ring and two from the large ring. The DNA locks can be unlocked to allow for bidirectional rotations through toehold-mediated strand displacement reactions.^44−47^ To enable optical characterizations of the rotary nanodevices, two fluorophores ATTO550 (blue) and ATTO647N (green) are tethered on the small and large rings, respectively. The fluorescence signal changes of the fluorophores, which strongly depend on their distances relative to the AuNPs, are used to optically report the rotation dynamics of the rotary devices in real time.^48^

### AuNP Rolling along the Small Ring

To elucidate the functions of the modular components, we first examine the interaction between the AuNPs and the small ring. As shown in Figure 2a, 12 foothold rows in six pairs (coded 1–6) are antisymmetrically arranged along the outer circumference of the small ring. Initially, both AuNPs are attached to foothold rows (1 and 2) through DNA hybridization. The remaining foothold rows are deactivated using respective blocking strands. An overview transmission electron microscopy (TEM) image of the assembled structures at this state “r(I)” is shown in Figure 2b (see SI Figure S8 for the small rings without AuNPs). The averaged TEM image in the inset of Figure 2b (see also SI Figure S11) reveals a high homogeneity of the assembled structures.

“r(I)”–“r(VII)” in Figure 2c depict the different states along the rolling route. The exact angle change for each state can be found in SI Figure S4. To ensure that the 12 footholds all point out from the small ring, they are separated not evenly but by 21 or 11 base pairs due to the limited dimension of the small ring circumference. More specifically, the angle change associated with 11 base pairs is ∼17.5°, while the angle change associated with 21 base pairs is ∼32.5° (see SI Figure S4). Figure 2a schematically illustrates the foot–foothold interaction between the AuNPs and the small ring, which enables simultaneous rolling of the two AuNPs along the outer circumference of the small ring, transiting from “r(I)” to “r(II)”. The motion of the AuNPs is powered by DNA fuels through toehold-mediated strand displacement reactions.^44−47^ The rolling of the two AuNPs along the ring is cooperative and driven by the same set of DNA fuels. At “r(I)”, blocking strands 1 and releasing strands 3 are added simultaneously. The blocking strands detach the feet of the AuNPs from foothold row 1 and subsequently block it. This eliminates the back rolling of the AuNPs and thus imparts motion directionality. Meanwhile, releasing strands 3 activate foothold row 3 for AuNP binding. Subsequently, the AuNPs are bound to foothold rows 2 and 3, reaching “r(II)”. The rolling direction (clockwise or counterclockwise) is programmable and determined by the sequences of the DNA fuels added to the system. For instance, to introduce one clockwise rolling step from “r(I)”, blocking strands 2 and releasing strands 6 are added.

In order to optically resolve the different states during the rolling process, two fluorophores, ATTO550 (blue) and ATTO647N (green) are tethered between foothold rows 4 and 5 on the opposite sides of the ring. Rolling of the AuNPs along the ring gives rise to distance variations between the fluorophores and the AuNPs, thus leading to distance-dependent electromagnetic quenching of the fluorophores.^48^Figure 2d presents the experimental fluorescence results, recorded using the dual-wavelength time-scan function of a fluorescence spectrometer (Jasco-FP 8500). For ATTO550, the excitation and emission wavelengths are 554 and 576 nm, respectively. For ATTO647N, the excitation and emission wavelengths are 646 and 664 nm, respectively. As shown in Figure 2d, fluorescence signal changes can be evidently identified, while the AuNPs roll along the small ring, transiting among different states. For instance, from “r(I)” to “r(II)” the fluorescence signals of ATTO550 and ATTO647N both decrease due to stronger quenching effects, resulting from the distance decreases between the AuNPs and their adjacent fluorophores, that is, AuNP (brown) relative to ATTO550 (blue) and AuNP (golden) relative to ATTO647N (green).

The theoretical fluorescence results at different states are presented in Figure 2e. The fluorescence rate γ_fl_ of a fluorophore is given by the product of its quantum yield *q* and its excitation rate γ_exc_. The change of the fluorescence rate under the influence of the AuNPs can be written as
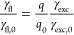
1where no subscript is used to indicate the quantities in the presence of the AuNPs, and the subscript ‘0’ denotes the corresponding quantities in free space.^49^ The ratio γ_exc_/γ_exc,0_ represents the excitation enhancement, which have been obtained from finite-element simulations of the near fields generated by a plane wave impinging onto the AuNPs at the wavelengths of 554 and 646 nm for ATTO550 and ATTO647N, respectively. The random orientations of structures in the solution are taken into account by averaging γ_exc_/γ_exc,0_ over all possible incidence directions and polarizations. The quantum yield *q* in eq 1 can be expressed as
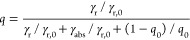
2in which γ_r_ represents the radiative decay rate in the presence of the AuNPs, γ_abs_ is the rate of energy absorption in the AuNPs, and γ_r,0_ denotes the radiative decay rate in free space.^49^ The factors γ_r_/γ_r,0_ and γ_abs_/γ_r,0_ are obtained from finite-element simulations of an emitting electric dipole placed next to the AuNP. The rotational freedom of the fluorophores attached to the DNA structures is taken into account by averaging γ_r_/γ_r,0_ and γ_abs_/γ_r,0_ over all possible dipole orientations. Furthermore, the fact that the fluorophores do not only emit at discrete wavelengths but over a spectral range is considered via averaging γ_r_/γ_r,0_ and γ_abs_/γ_r,0_ over their intrinsic emission spectra. For AuNPs as small as 15 nm, the dominating effect is absorption, resulting in quenching of the fluorescence, when the fluorophores approach the AuNPs. As shown in Figure 2d,e, the experimental and theoretical results agree qualitatively well. The deviations are likely due to the sample imperfections. To demonstrate the bidirectionality of the rotary devices, SI Figure S13 presents the experimental and theoretical results for programmable switching between clockwise and counterclockwise rolling of the AuNPs along the small ring.

### AuNP Rolling along the Large Ring

Next, we investigate the interaction between the AuNPs and the large ring. As shown in Figure 3a, 12 foothold rows in six pairs (coded 1–6) are antisymmetrically arranged along the inner circumference of the large ring. At “R(I)”, the two AuNPs are oppositely attached to the ring via foothold rows (1 and 2) through DNA hybridization. ATTO550 (blue) and ATTO647N (green) are tethered between foothold rows (4 and 5) on the one side and (3 and 4) on the other side, respectively. The overview and averaged TEM images in Figure 3b reveal high quality of the assembled structures.

The stepwise rolling of the AuNPs along the large ring is powered by DNA fuels through toehold-mediated strand displacement reactions. Figure 3a depicts one counterclockwise rolling step from “R(I)” to “R(II)” upon simultaneous addition of blocking strands 1 and releasing strands 3. Figure 3c illustrates the successive rolling of the AuNPs, transiting among different states from “R(I)” to “R(VII)”. The exact angle change for each state can be found in SI Figure S3. Figure 3d,e present the experimental and theoretical fluorescence results recorded during the rolling process, which agree qualitatively well. From “R(V)” to “R(VII)”, the fluorescence changes of ATTO647N are not very distinct, because at these states its spacings to the two AuNPs are relatively large, resulting in weak quenching effects. For this reason, we have always tracked the fluorescence changes of both fluorophores, so that if one fluorophore loses its distance sensitivity, the information on the rotation dynamics can be analyzed using the other fluorophore (e.g., ATTO550). It is also possible to position a third fluorophore emitting at a different wavelength on the device to cover the distance insensitive region of ATTO647N, at a price of complex optical characterizations. Additional experimental data, which demonstrate the reversibility of the rotary devices, is presented in SI Figure S19.

### AuNP Sliding in between Two Rings

The planetary gearset nanodevice is formed following the assembly process as shown in Figure 1b. The correct orientation and relative position of the two rings are imposed by the three locks and further stabilized by the two AuNPs intercalated between the rings. SI Figures S21 and S22 present the TEM images of the assembled double rings without the AuNPs. The role of the AuNPs that substantially helps to enhance the structural stability can be clearly appreciated. In the planetary gearset nanodevice as shown in Figure 4a, ATTO550 (blue) is tethered on the small ring between foothold rows 4 and 5, while ATTO647N (green) is attached to the large ring between the remote foothold rows 3 and 4.

The small and large rings are both decorated with 12 footholds. The foothold rows (coded 1–6) within each ring are antisymmetrically arranged as discussed in Figures 2 and 3. In addition, the arrangement of these footholds along the small and large rings is also antisymmetric with respect to one another. Due to such an arrangement, each AuNP can always bind to four foothold rows at each step, two from each ring, during rolling. For instance, at “I” the AuNP (brown) is attached to foothold rows 3 and 4 extended from the large ring and the corresponding foothold rows 4 and 3 from the small ring. Meanwhile, the other AuNP (golden) is attached to the same four foothold rows. To enable the dynamic process, unlocking strands (see SI Table S15) are first added to open the three locks through toehold-mediated strand displacement reactions. Next, upon addition of the same set of DNA fuels, the two AuNPs can be simultaneously driven to roll in between the two rings. This introduces sliding of the rings along opposite directions. As illustrated in the enlarged section in Figure 4a, the addition of blocking strands 3 and releasing strands 5 results in the dissociation of the AuNP feet from foothold row 3 on both rings and the subsequent binding of the AuNP feet to foothold row 5 on both rings. The two AuNPs are then bound to foothold rows 4 and 5. The nanodevice thus reaches the next state, “II”.

The function of the AuNPs in our nanodevice resembles that of the planet gears in a planetary gearset, which slide the sun and ring gears as pinions along opposite directions. Such a movement scheme mediated by the AuNPs gives rise to a relative angle change between the two rings, which is twice larger than that of the AuNPs for each step. This relative angle change is highlighted using the pink arrow on the large ring in Figure 4a, while the small ring is fixed in position. The overview and averaged TEM images of the assembled rotary devices are shown in Figure 4b (see also SI Figures S23 and S24). As a comparison, the TEM images of the structures with 10 nm AuNPs as pinion gears in a less tightly fitted geometry are shown in SI Figures S25 and S26.

Figure 4c illustrates the rotation steps of the planetary gearset nanodevice, when it transits from state “I” to state “VII”. As the relative angle change between the two rings are doubled for each step, in total the large ring has rotated against the small ring by 360°, after the system reaches state “VII”. Figure 4d presents the experimental fluorescence results, which show a qualitative agreement with the calculated results in Figure 4e. The deviations between the experimental and theoretical results could be attributed to structural imperfections. As shown by the TEM images taken after the rotation process in SI Figure S27, some double ring structures exhibit missing AuNPs, which can result in weaker quenching effects to the fluorophores. This likely leads to the higher fluorescence signals at state VII, when compared to those at state I in the experiment. The structural and sequence designs can be further optimized to enhance the device performance and fidelity. To further validate the rotation mechanism of the planetary gearset nanodevice, two smaller AuNPs (5 nm) are immobilized on the small and large rings as markers, respectively. The TEM images of the representative states clearly prove the designated relative movements among the AuNPs (see SI Figures S29 to S32).

## Conclusions

One of the exciting directions in DNA nanotechnology is to accomplish functional synthetic nanomachines, in which modular components can be assembled together to work in concert. Inspirations for tackling this challenge can be found in biology. Learning from the design models and working principles of biological machines can be very instructive to build, control, and program artificial machinery on the nanoscale. We have demonstrated the modular assembly of a structurally well-defined nanodevice, which can carry out programmable rotary motions powered by DNA fuels. The discrete rotation steps of the device have been optically reported using fluorescence spectroscopy in real time. The experimental results are well supported by the theoretical calculations. Due to the modularity of our design, other functional components, such as a DNA-based extending filament, can be attached to the concentric origami rings in analogy to the linking of the flagellar filament and the basal body in a bacterial flagellum. As a result, the nanoscopic operation of the molecular device with precisely programmed functions could be transduced and upscaled to a microscopic object. Our concept could be also insightful for the realization of advanced artificial nanofactories assembled from modular artificial machines, which are tightly orchestrated to execute complex tasks or yield useful biochemical products.^42^

## Methods and Experiments

### DNA Origami Assembly

DNA staple strands and DNA fuel (blocking and releasing) strands were purchased from Sigma-Aldrich (https://www.sigmaaldrich.com). The DNA scaffolds (p7249 and p8064) were purchased from Eurofins Genomics (Aldrich (https://www.eurofins.com)). The small origami rings were assembled from 20 nM p7249 scaffold strands, 200 nM of each set of the staple strands (core staples, locks and fluorophore-modified DNA strands; 10-fold excess) and 1 μM of appropriate blocking strands to deactivate the footholds (50-fold excess) in a 1 × TE buffer (40 mM Tris, 2 mM EDTA, pH 8) with 20 mM MgCl_2_. For different initial states, different sets of footholds were blocked to prepare the rings for the assembly of the rotary devices. The assembly mixture was annealed in a thermal cycler using a 20 h annealing program (85 °C for 5 min, 70–61 °C with −1 °C/min, 60–51 °C with −1 °C/1 h, 50–22 °C with −1 °C/20 min, and 15 °C hold). The large origami rings were assembled in a similar way. More specifically, the structures were folded using the p8064 scaffold strands and the assembly buffer contained 1 × TE (40 mM Tris, 2 mM EDTA, pH 8) with 14 mM MgCl_2_. The molar ratios of scaffolds to DNA staples and the annealing program were the same as those for the small origami rings. Successfully assembled origami structures were subjected to agarose gel electrophoresis for purification (see section ‘Agarose gel electrophoresis’).

### Surface Modification of the AuNPs with BSPP

AuNPs (15 nm) were purchased from Sigma-Aldrich Aldrich (https://www.sigmaaldrich.com). BSPP (3.75 mg) was added to the Au colloidal solution (5 mL, OD ∼ 1) and the mixture was shaken overnight at room temperature. The AuNP–BSPP solution was centrifuged at 8000*g* for 45 min, the supernatant was discharged and the AuNPs were resuspended in a 250 μL BSPP solution (2.5 mM). The concentration of the AuNPs was estimated according to the optical absorption at 520 nm.

### Preparation of the AuNP–DNA Conjugates

Thiol-modified DNA strands were purchased from Sigma-Aldrich (https://www.sigmaaldrich.com). AuNP–DNA conjugation was accomplished according to Hao et al.^50^ with minor modifications. The disulfide bond in the thiol-modified oligonucleotides was reduced using tris(2-carboxythyl)phosphine (TCEP, 100 mM, 1 h) in water. Reduced thiol-modified oligonucleotides and BSPP-coated AuNPs were subsequently mixed at a molar ratio of 1000:1 to reach a final volume of 100 μL. This solution was immediately combined with 900 μL of *n*-Butanol, followed by a quick vortex mixing for several seconds. 100 μL of 0.5 × TBE buffer (45 mM Tris, 45 mM Boric acid, 1 mM EDTA) was added to the solution, followed by a second quick vortex mixing and centrifugation at 2000*g* for several seconds to facilitate the phase separation. DNA-functionalized AuNPs were recovered as the sublayer of the two liquid phases. To remove the extra free oligonucleotides, the AuNP–DNA conjugates were washed three times at 8000*g* for 45 min using a 0.5 × TBE buffer. The concentration of the AuNP–DNA conjugates was estimated according to the optical absorption at 520 nm.

### Self-Assembly of the AuNPs on the DNA Origami Structures

The origami structures with active footholds 1 and 2 (characterization of the small and large origami rings) and structures with active footholds 3 and 4 (characterization of the entire rotary devices) were used, respectively. The remaining footholds were blocked. The DNA-coated and purified AuNPs were added to the respective structures in an excess of 10 AuNPs per binding site on the origami. The mixture was annealed in a thermo cycler for 24 h at 23 °C. Successfully assembled AuNP-functionalized origami structures were purified using agarose gel electrophoresis (see the “Agarose Gel Electrophoresis” section).

### Self-Assembly of the Planetary Gearset Structures

The rotary nanodevice was assembled from the small origami ring with active footholds 3 and 4 and the AuNP-functionalized large origami ring with AuNPs attached to footholds 3 and 4. Both structures were equipped with the locking strands. The two components were mixed in a molar ratio of 1:1 in a 0.5 × TBE buffer with 11 mM MgCl_2_. For assembly, a thermo cycler with a linear temperature ramp from 30 to 20 °C (30 min/°C) for 5 cycles was used. Successfully assembled structures were purified using agarose gel electrophoresis (see the “Agarose Gel Electrophoresis” section).

### Agarose Gel Electrophoresis

The origami structures and the AuNP-decorated origami structures were subjected to agarose gel electrophoresis for purification. Samples were run in a 0.7% agarose gel in a 0.5 × TBE-Mg^2+^ buffer (45 mM Tris, 45 mM Boric acid, 1 mM EDTA, 11 mM Mg^2+^) for 3 h at 8 V/cm in a gel box immersed in an ice–water bath. SYBR gold was used to stain the origami structures. After segregating the gel bands, the origami structures were extracted by squeezing and being isolated using Quantum Prep Squeeze ’N Freeze filter units (Bio-Rad Laboratories, Inc., Hercules, CA) at 500 × g for 1 min. The concentration of the purified origami structures was estimated according to the optical absorption at 260 nm. Estimation of the concentration of the AuNP-modified origami structures was accomplished by measuring the optical absorption of the AuNPs at 520 nm.

### Transmission Electron Microscopy

Uranyl formate for negative staining was purchased from Polysciences, Inc. DNA origami structures and the AuNP-decorated origami structures were imaged using Philips CM 200 TEM operating at 200 kV. For imaging, negatively stained samples were prepared on freshly glow-discharged carbon/Formvar FCF 400-CU TEM grids (Electron Microscopy Sciences, Hatfield, PA). The sample solution was adsorbed on the grid and subsequently stained with 2% aqueous uranyl formate solution containing 17.5 mM sodium hydroxide. Samples were dried overnight. Data processing was performed using the Fiji for ImageJ software.^51^ Average TEM images were obtained using the EMAN2 software.^52^

### Fluorescence Spectroscopy

Fluorescence spectra were measured using a Jasco-FP8500 fluorescence spectrometer with a quartz SUPRASIL ultramicro cuvette (path length, 10 mm). All measurements were carried out at room temperature in a 0.5 × TBE buffer with 11 mM MgCl_2_ at pH 8 after agarose gel purification. For the *in situ* fluorescence measurements of the AuNP rolling along the small and large DNA origami rings, a 100 μL solution containing ∼3 nM of the structures at the initial configuration was used. For the measurements of the rotary devices, a 100 μL solution containing ∼0.5–1 nM of the structures at the initial configuration was used. The fluorescence emissions at 576 and 664 nm were tracked in real time using the dual-wavelength time-scan acquisition mode and a data pitch of 5s. The excitation wavelengths were 554 and 646 nm, respectively. Appropriate strands to open the locks were added to enable the rotation. Respective blocking and releasing strands were subsequently added to enable the programmed rotations (∼300 times excess).

### Theoretical Calculations

The finite-element simulations were performed using commercially available software COMSOL Multiphysics. The dielectric function of Au was interpolated from experimental data.^53^ Water as the surrounding medium was taken into account with a refractive index of 1.332. The emission spectra of the fluorophores, as well as the intrinsic quantum yields *q*_0_ (0.8 for ATTO 550 and 0.65 for ATTO 647N) were considered as specified by the supplier of the molecules (https://www.atto-tec.com).

## Supporting Information Available

Design details of the DNA origami and the planetary gearset nanodevice as well as additional results, such as TEM images and fluorescence data (PDF)

## References

[ref1] Guo P.; Noji H.; Yengo C. M.; Zhao Z.; Grainge I. Biological Nanomotors with a Revolution, Linear, or Rotation Motion Mechanism. Microbiol. Mol. Biol. Rev. 2016, 80 (1), 161–86. 10.1128/MMBR.00056-15. 26819321 PMC4771369

[ref2] Abrahams J. P.; Leslie A. G.; Lutter R.; Walker J. E. Structure at 2.8 A Resolution of F1-Atpase from Bovine Heart Mitochondria. Nature 1994, 370 (6491), 621–8. 10.1038/370621a0. 8065448

[ref3] Boyer P. D. The ATP Synthase -A Splendid Molecular Machine. Annu. Rev. Biochem. 1997, 66, 717–49. 10.1146/annurev.biochem.66.1.717. 9242922

[ref4] Stock D.; Leslie A. G.; Walker J. E. Molecular Architecture of the Rotary Motor in ATP Synthase. Science 1999, 286 (5445), 1700–5. 10.1126/science.286.5445.1700. 10576729

[ref5] Okuno D.; Iino R.; Noji H. Rotation and Structure of Fof1-ATP Synthase. J. Biochem. 2011, 149 (6), 655–64. 10.1093/jb/mvr049. 21524994

[ref6] Sambongi Y.; Iko Y.; Tanabe M.; Omote H.; Iwamoto-Kihara A.; Ueda I.; Yanagida T.; Wada Y.; Futai M. Mechanical Rotation of the C Subunit Oligomer in ATP Synthase (F0F1): Direct Observation. Science 1999, 286 (5445), 1722–4. 10.1126/science.286.5445.1722. 10576736

[ref7] Berg H. C. Bacterial Flagellar Motor. Curr. Biol. 2008, 18 (16), R689–91. 10.1016/j.cub.2008.07.015. 18727898

[ref8] Samatey F. A.; Imada K.; Nagashima S.; Vonderviszt F.; Kumasaka T.; Yamamoto M.; Namba K. Structure of the Bacterial Flagellar Protofilament and Implications for a Switch for Supercoiling. Nature 2001, 410 (6826), 331–7. 10.1038/35066504. 11268201

[ref9] Xue R. D.; Ma Q.; Baker M. A. B.; Bai F., A Delicate Nanoscale Motor Made by Nature-The Bacterial Flagellar Motor. Adv. Sci. 2015, 2 ( (9), ).1500129 10.1002/advs.201500129 PMC5115386 27980978

[ref10] Yonekura K.; Maki-Yonekura S.; Namba K. Complete Atomic Model of the Bacterial Flagellar Filament by Electron Cryomicroscopy. Nature 2003, 424 (6949), 643–50. 10.1038/nature01830. 12904785

[ref11] Chang Y.; Zhang K.; Carroll B. L.; Zhao X.; Charon N. W.; Norris S. J.; Motaleb M. A.; Li C.; Liu J. Molecular Mechanism for Rotational Switching of the Bacterial Flagellar Motor. Nat. Struct. Mol. Biol. 2020, 27 (11), 1041–1047. 10.1038/s41594-020-0497-2. 32895555 PMC8129871

[ref12] Lee L. K.; Ginsburg M. A.; Crovace C.; Donohoe M.; Stock D. Structure of the Torque Ring of the Flagellar Motor and the Molecular Basis for Rotational Switching. Nature 2010, 466 (7309), 996–1000. 10.1038/nature09300. 20676082 PMC3159035

[ref13] Minamino T.; Imada K.; Kinoshita M.; Nakamura S.; Morimoto Y. V.; Namba K. Structural Insight into the Rotational Switching Mechanism of the Bacterial Flagellar Motor. Plos Biol. 2011, 9 (5), E1000616 10.1371/journal.pbio.1000616. 21572987 PMC3091841

[ref14] Deme J. C.; Johnson S.; Vickery O.; Muellbauer A.; Monkhouse H.; Griffiths T.; James R. H.; Berks B. C.; Coulton J. W.; Stansfeld P. J.; Lea S. M. Structures of the Stator Complex that Drives Rotation of the Bacterial Flagellum. Nat. Microbiol 2020, 5 (12), 1553 10.1038/s41564-020-0788-8. 32929189 PMC7610383

[ref15] Santiveri M.; Roa-Eguiara A.; Kuhne C.; Wadhwa N.; Hu H.; Berg H. C.; Erhardt M.; Taylor N. M. I. Structure and Function of Stator Units of the Bacterial Flagellar Motor. Cell 2020, 183 (1), 244–257. E16 10.1016/j.cell.2020.08.016. 32931735

[ref16] Rothemund P. W. Folding DNA to Create Nanoscale Shapes and Patterns. Nature 2006, 440 (7082), 297–302. 10.1038/nature04586. 16541064

[ref17] Douglas S. M.; Dietz H.; Liedl T.; Hogberg B.; Graf F.; Shih W. M. Self-Assembly of DNA into Nanoscale Three-Dimensional Shapes. Nature 2009, 459 (7245), 414–8. 10.1038/nature08016. 19458720 PMC2688462

[ref18] Dietz H.; Douglas S. M.; Shih W. M. Folding DNA into Twisted and Curved Nanoscale Shapes. Science 2009, 325 (5941), 725–30. 10.1126/science.1174251. 19661424 PMC2737683

[ref19] Douglas S. M.; Marblestone A. H.; Teerapittayanon S.; Vazquez A.; Church G. M.; Shih W. M. Rapid Prototyping of 3D DNA-Origami Shapes with Cadnano. Nucleic Acids Res. 2009, 37 (15), 5001–6. 10.1093/nar/gkp436. 19531737 PMC2731887

[ref20] Ke Y.; Douglas S. M.; Liu M.; Sharma J.; Cheng A.; Leung A.; Liu Y.; Shih W. M.; Yan H. Multilayer DNA Origami Packed on a Square Lattice. J. Am. Chem. Soc. 2009, 131 (43), 15903–8. 10.1021/ja906381y. 19807088 PMC2821935

[ref21] Pan K.; Kim D. N.; Zhang F.; Adendorff M. R.; Yan H.; Bathe M. Lattice-Free Prediction of Three-Dimensional Structure of Programmed DNA Assemblies. Nat. Commun. 2014, 5, 5578 10.1038/ncomms6578. 25470497 PMC4268701

[ref22] Jones M. R.; Seeman N. C.; Mirkin C. A. Nanomaterials. Programmable Materials and the Nature of the DNA Bond. Science 2015, 347 (6224), 1260901 10.1126/science.1260901. 25700524

[ref23] Chen Y.; Wang M. S.; Mao C. D. An Autonomous DNA Nanomotor Powered by a DNA Enzyme. Angew. Chem. Int. Edit 2004, 43 (27), 3554–3557. 10.1002/anie.200453779. 15293243

[ref24] Yin P.; Yan H.; Daniell X. G.; Turberfield A. J.; Reif J. H. A Unidirectional DNA Walker that Moves Autonomously along a Track. Angew. Chem. Int. Edit 2004, 43 (37), 4906–4911. 10.1002/anie.200460522. 15372637

[ref25] Omabegho T.; Sha R.; Seeman N. C. A Bipedal DNA Brownian Motor with Coordinated Legs. Science 2009, 324 (5923), 67–71. 10.1126/science.1170336. 19342582 PMC3470906

[ref26] Lund K.; Manzo A. J.; Dabby N.; Michelotti N.; Johnson-Buck A.; Nangreave J.; Taylor S.; Pei R. J.; Stojanovic M. N.; Walter N. G.; Winfree E.; Yan H. Molecular Robots Guided by Prescriptive Landscapes. Nature 2010, 465 (7295), 206–210. 10.1038/nature09012. 20463735 PMC2907518

[ref27] Urban M. J.; Zhou C.; Duan X. Y.; Liu N. Optically Resolving the Dynamic Walking of a Plasmonic Walker Couple. Nano Lett. 2015, 15 (12), 8392–8396. 10.1021/acs.nanolett.5b04270. 26571209

[ref28] Zhou C.; Duan X. Y.; Liu N., A Plasmonic Nanorod that Walks on DNA Origami. Nat. Commun. 2015, 6. 10.1038/ncomms9102 PMC4560816 26303016

[ref29] Marras A. E.; Zhou L.; Su H. J.; Castro C. E. Programmable Motion of DNA Origami Mechanisms. Proc. Natl. Acad. Sci. U S A 2015, 112 (3), 713–8. 10.1073/pnas.1408869112. 25561550 PMC4311804

[ref30] List J.; Falgenhauer E.; Kopperger E.; Pardatscher G.; Simmel F. C. Long-Range Movement of Large Mechanically Interlocked DNA Nanostructures. Nat. Commun. 2016, 7, 12414 10.1038/ncomms12414. 27492061 PMC4980458

[ref31] Urban M. J.; Both S.; Zhou C.; Kuzyk A.; Lindfors K.; Weiss T.; Liu N., Gold Nanocrystal-Mediated Sliding of Doublet DNA Origami Filaments. Nat. Commun. 2018, 9. 10.1038/s41467-018-03882-w PMC5899135 29654323

[ref32] Zhan P.; Both S.; Weiss T.; Liu N. DNA-Assembled Multilayer Sliding Nanosystems. Nano Lett. 2019, 19 (9), 6385–6390. 10.1021/acs.nanolett.9b02565. 31438681 PMC6746187

[ref33] Zhan P.; Urban M. J.; Both S.; Duan X.; Kuzyk A.; Weiss T.; Liu N. DNA-Assembled Nanoarchitectures with Multiple Components in Regulated and Coordinated Motion. Sci. Adv. 2019, 5 (11), Eaax6023 10.1126/sciadv.aax6023. 31819901 PMC6884410

[ref34] Ketterer P.; Willner E. M.; Dietz H. Nanoscale Rotary Apparatus Formed from Tight-Fitting 3D DNA Components. Sci. Adv. 2016, 2 (2), E1501209 10.1126/sciadv.1501209. 26989778 PMC4788491

[ref35] Tomaru T.; Suzuki Y.; Kawamata I.; Nomura S. M.; Murata S. Stepping Operation of a Rotary DNA Origami Device. Chem. Commun. (Camb) 2017, 53 (55), 7716–7719. 10.1039/C7CC03214E. 28548145

[ref36] Kopperger E.; List J.; Madhira S.; Rothfischer F.; Lamb D. C.; Simmel F. C. A Self-Assembled Nanoscale Robotic Arm Controlled by Electric Fields. Science 2018, 359 (6373), 296–300. 10.1126/science.aao4284. 29348232

[ref37] Lauback S.; Mattioli K. R.; Marras A. E.; Armstrong M.; Rudibaugh T. P.; Sooryakumar R.; Castro C. E. Real-Time Magnetic Actuation of DNA Nanodevices *via* Modular Integration With Stiff Micro-Levers. Nat. Commun. 2018, 9 (1), 1446 10.1038/s41467-018-03601-5. 29654315 PMC5899095

[ref38] Xin L.; Zhou C.; Duan X.; Liu N. A Rotary Plasmonic Nanoclock. Nat. Commun. 2019, 10 (1), 5394 10.1038/s41467-019-13444-3. 31776340 PMC6881389

[ref39] Yang Y.; Zhang S.; Yao S.; Pan R.; Hidaka K.; Emura T.; Fan C.; Sugiyama H.; Xu Y.; Endo M.; Qian X. Programming Rotary Motions with a Hexagonal DNA Nanomachine. Chemistry 2019, 25 (20), 5158–5162. 10.1002/chem.201900221. 30791173

[ref40] Xin L.; Duan X.; Liu N. Dimerization And Oligomerization Of DNA-Assembled Building Blocks for Controlled Multi-Motion in High-Order Architectures. Nat. Commun. 2021, 12 (1), 3207 10.1038/s41467-021-23532-y. 34050157 PMC8163789

[ref41] Ahmadi Y.; Nord A. L.; Wilson A. J.; Hutter C.; Schroeder F.; Beeby M.; Barisic I. The Brownian and Flow-Driven Rotational Dynamics of a Multicomponent DNA Origami-Based Rotor. Small 2020, 16 (22), E2001855 10.1002/smll.202001855. 32363713

[ref42] Simmel F. C. DNA-Based Assembly Lines and Nanofactories. Curr. Opin. Biotechnol. 2012, 23 (4), 516–21. 10.1016/j.copbio.2011.12.024. 22237015

[ref43] Thubagere A. J.; Li W.; Johnson R. F.; Chen Z.; Doroudi S.; Lee Y. L.; Izatt G.; Wittman S.; Srinivas N.; Woods D.; Winfree E.; Qian L., A Cargo-Sorting DNA Robot. Science 2017, 357 ( (6356), ). 10.1126/science.aan6558 28912216

[ref44] Zhang D. Y.; Seelig G. Dynamic DNA Nanotechnology Using Strand-Displacement Reactions. Nat. Chem. 2011, 3 (2), 103–13. 10.1038/nchem.957. 21258382

[ref45] Zhang D. Y.; Winfree E. Control of DNA Strand Displacement Kinetics Using Toehold Exchange. J. Am. Chem. Soc. 2009, 131 (47), 17303–14. 10.1021/ja906987s. 19894722

[ref46] Srinivas N.; Ouldridge T. E.; Sulc P.; Schaeffer J. M.; Yurke B.; Louis A. A.; Doye J. P.; Winfree E. On The Biophysics And Kinetics of Toehold-Mediated DNA Strand Displacement. Nucleic Acids Res. 2013, 41 (22), 10641–58. 10.1093/nar/gkt801. 24019238 PMC3905871

[ref47] Zhang J. X.; Fang J. Z.; Duan W.; Wu L. R.; Zhang A. W.; Dalchau N.; Yordanov B.; Petersen R.; Phillips A.; Zhang D. Y. Predicting DNA Hybridization Kinetics from Sequence. Nat. Chem. 2018, 10 (1), 91–98. 10.1038/nchem.2877. 29256499 PMC5739081

[ref48] Dulkeith E.; Morteani A. C.; Niedereichholz T.; Klar T. A.; Feldmann J.; Levi S. A.; Van Veggel F. C.; Reinhoudt D. N.; M?ller M.; Gittins D. I. Fluorescence Quenching of Dye Molecules Near Gold Nanoparticles: Radiative and Nonradiative Effects. Phys. Rev. Lett. 2002, 89 (20), 203002 10.1103/PhysRevLett.89.203002. 12443474

[ref49] Bharadwaj P.; Novotny L. Spectral Dependence of Single Molecule Fluorescence Enhancement. Opt. Express 2007, 15 (21), 14266–74. 10.1364/OE.15.014266. 19550702

[ref50] Hao Y.; Li Y.; Song L.; Deng Z. Flash Synthesis of Spherical Nucleic Acids with Record DNA Density. J. Am. Chem. Soc. 2021, 143 (8), 3065–3069. 10.1021/jacs.1c00568. 33599474

[ref51] Schindelin J.; Arganda-Carreras I.; Frise E.; Kaynig V.; Longair M.; Pietzsch T.; Preibisch S.; Rueden C.; Saalfeld S.; Schmid B.; Tinevez J. Y.; White D. J.; Hartenstein V.; Eliceiri K.; Tomancak P.; Cardona A. Fiji: An Open-Source Platform for Biological-Image Analysis. Nat. Methods 2012, 9 (7), 676–82. 10.1038/nmeth.2019. 22743772 PMC3855844

[ref52] Tang G.; Peng L.; Baldwin P. R.; Mann D. S.; Jiang W.; Rees I.; Ludtke S. J. EMAN2: An Extensible Image Processing Suite for Electron Microscopy. J. Struct. Biol. 2007, 157 (1), 38–46. 10.1016/j.jsb.2006.05.009. 16859925

[ref53] Johnson P. B., Christy R. W. Optical Constants ofthe Noble Metals. Phys. Rev. B 1972.6 4370 10.1103/PhysRevB.6.4370

