# Supplementary material for: DNA Assembly of Modular Components into a Rotary Nanodevice
Source: ACS Nano. 2022 Mar 14;16(4):5284–91. doi: 10.1021/acsnano.1c10160 (PMC9047004; doi:10.1021/acsnano.1c10160)
Supplement: Supplementary file 1 — nn1c10160_si_001.pdf [file nn1c10160_si_001.pdf]

Supporting Information

DNA Assembly of Modular Components into a Rotary Nanodevice

Andreas Peil, [†,‡] *Ling Xin,* [†,‡*] *Steffen Both,* [§] *Luyao Shen,* [||] *Yonggang Ke,* [||] *Thomas Weiss,* [§⊥] *Pengfei Zhan,* [†,‡*] *and Na Liu* [†,‡*]

[†] 2nd Physics Institute, University of Stuttgart, Pfaffenwaldring 57, 70569 Stuttgart, Germany

[‡] Max Planck Institute for Solid State Research, Heisenbergstrasse 1, 70569 Stuttgart, Germany

[§] 4th Physics Institute, University of Stuttgart, Pfaffenwaldring 57, 70569 Stuttgart, Germany

[||] Wallace L. Coulter Department of Biomedical Engineering, Emory University and Georgia Institute of Technology, Atlanta, GA, 30322 USA

[⊥] Institute of Physics, University of Graz, and NAWI Graz, Universitätsplatz 5, 8010 Graz, Austria

Content

I. **DNA origami design**

II. **Supplementary Figures**

III. **DNA sequences**

IV. **References**

I. DNA origami design

Small origami ring. The small ring structure was designed using the caDNAno software.[1] It consists of a 10-helix bundle arranged in a 'honeycomb' lattice. Constant bending of the DNA bundle was achieved by the deletion and addition of base pairs along the helices. The DNA bundle was split into four parts. Helices of the innermost part (helices 3 and 9, 1st layer) have 44 deletions, helices of the 2nd layer (helices 2, 4 and 8) have 22 deletions, helices of the 3rd layer (helices 1, 5 and 7) have 22 insertions and helices of the 4th layer (helices 0 and 6) have 44 insertions. To fold the structure, the p7249 scaffold was used. Due to the small size of the origami, only 2309 bases were used. To prevent the remaining scaffold to disturb the rotation process, the unused part of the scaffold strand points towards the center of the ring. At the end of the DNA bundle, scaffold and staple strand overhangs connect each head and tail region to ensure a closed ring geometry (for more details see Supplementary Figure S1).

Large origami ring. The large ring structure was designed with caDNAno[1] software using the p8064 scaffold. The large ring consists of a 12-helix bundle arranged in a 'honeycomb' lattice. Constant bending of the DNA bundles was achieved by the deletion and insertion of base pairs along the helices. The DNA bundle was split into six parts. Helices of the innermost part (helices 0 and 11, 1st layer) have 90 deletions, helices of the 2nd (helices 1 and 10) and 3rd part (helices 2 and 9) have 54 and 18 deletions, respectively, helices of the 4th (helices 3 and 8), 5th (helices 4 and 7) and 6th layer (helices 5 and 6) have 18, 54 and 90 insertions, accordingly. At the end of the DNA bundle, staple strand overhangs connect each head and tail region to ensure a closed ring geometry (for more details see Supplementary Figure S2).

II. Supplementary Figures

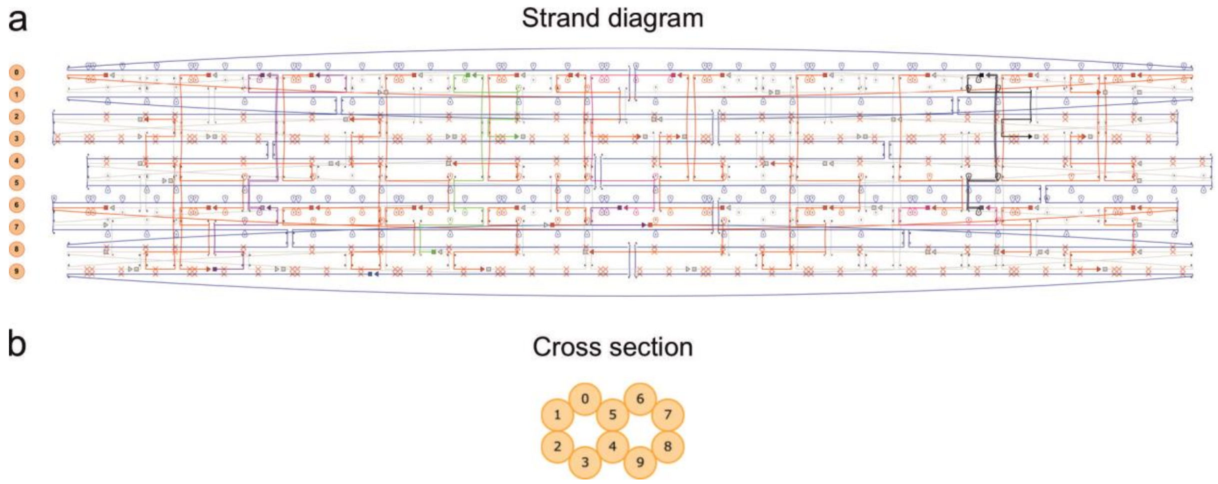

Supplementary Figure S1. (a) caDNAno design and strand routing for the small DNA origami ring. Blue: p7249 scaffold; light gray: core staples; red: footholds; green: ATTO550 staples; black: ATTO647N staples; purple: locking strands; pink: foothold strands with locking region at the 3’ end. According to whether only the small DNA origami ring or the rotary device was assembled, addition of ATTO647 and locks (black and pink staples) had to be adjusted. (b) Cross section of the DNA bundle layout.

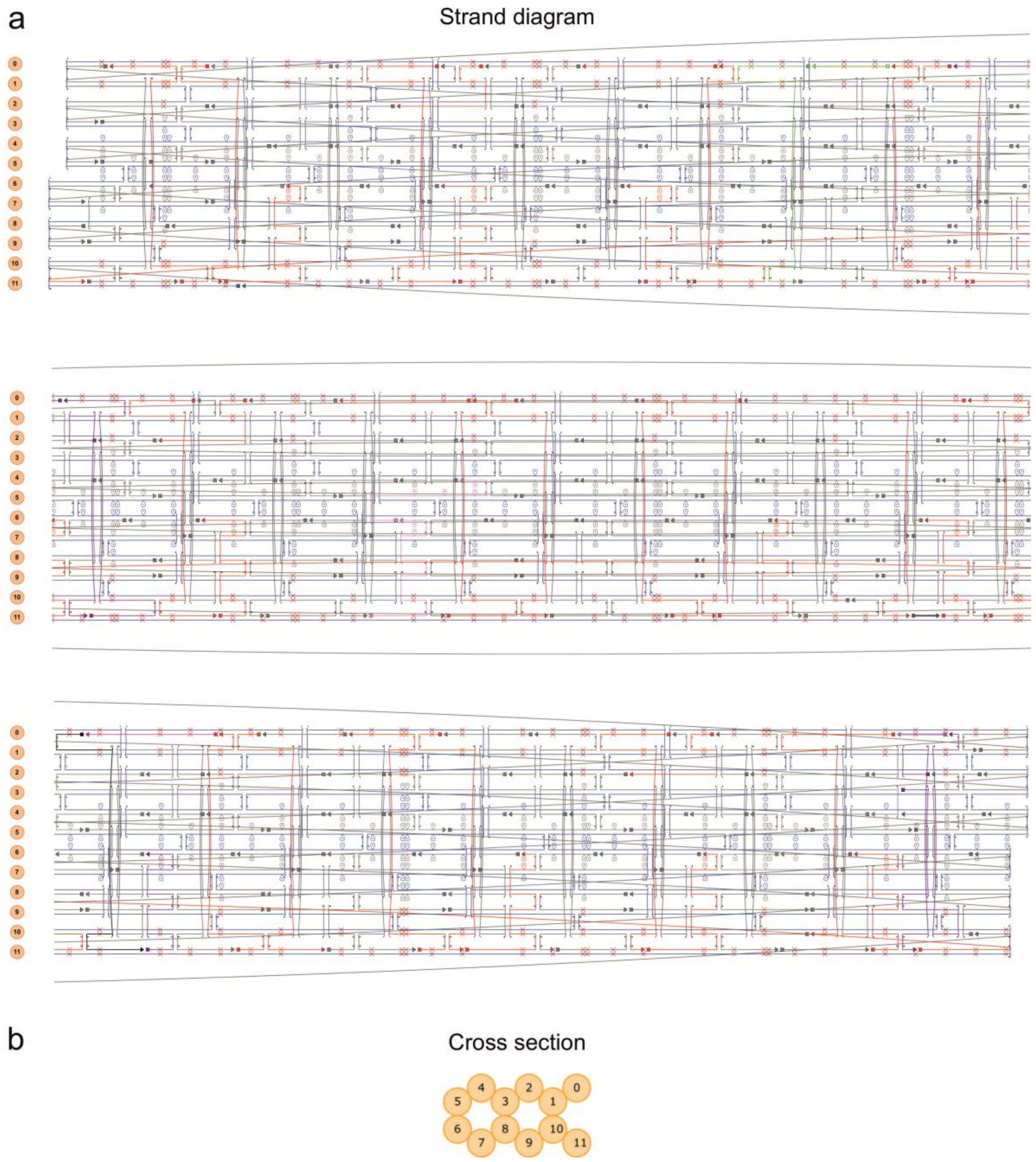

Supplementary Figure S2. (a) caDNAno design and strand routing for the large DNA origami ring. Blue: p8064 scaffold; light gray: core staples; red: footholds; green: ATTO647N staples; black: ATTO550 position and locking strand, respectively; purple: locking strands; pink: foothold strands with locking region or ATTO550 hybridization region at the 3' end. According to whether only the large DNA origami ring or the rotary device was assembled, addition of ATTO550 staples and locks (black and pink staples) had to be adjusted. (b) Cross section of the DNA bundle layout.

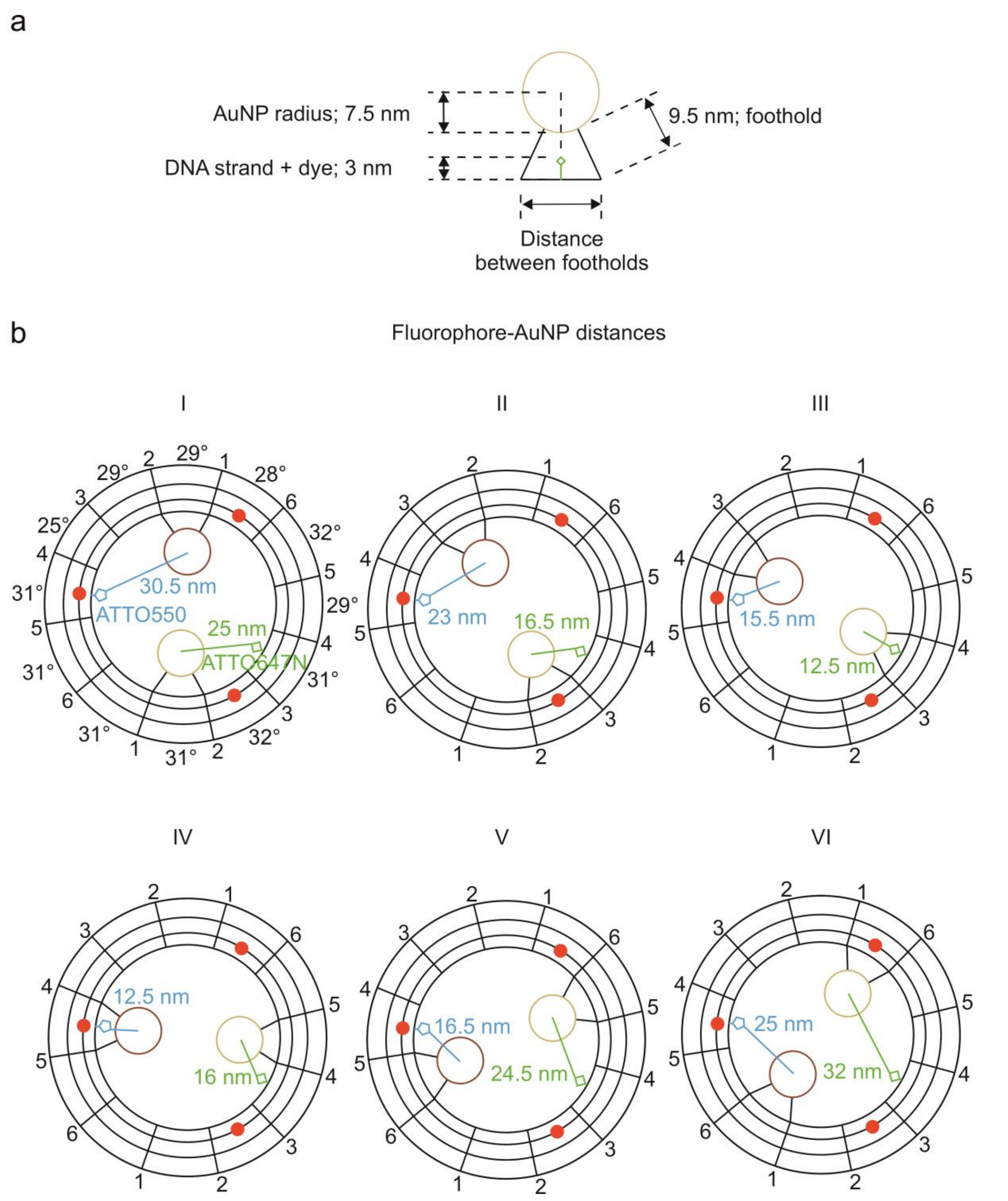

Supplementary Figure S3. (a) Illustration of the basic distances. The red dots represent the three locking strand positions. (b) Angles between the foothold rows on the large origami ring and the fluorophore-AuNP distances at different states. The fluorophore-AuNP distances relevant for the theoretical calculations can be found in Supplementary Table S4.

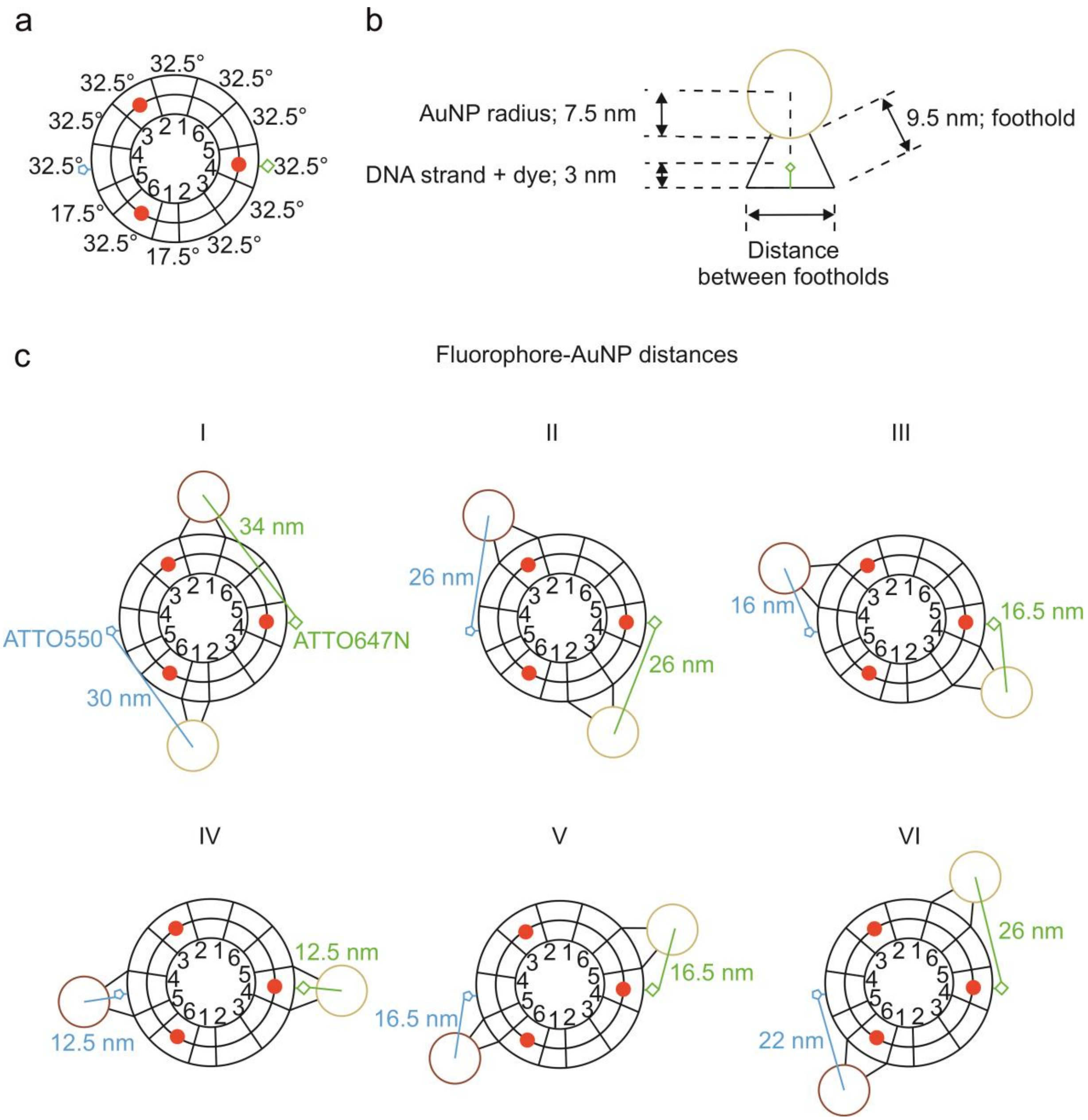

Supplementary Figure S4. (a) Angles between the foothold rows on the small origami ring. The positions of the locks are indicated with red dots. (b) Illustration of the basic distances. (c) Fluorophore-AuNP distances at different states. The fluorophore-AuNP distances relevant for the theoretical calculations can be found in Supplementary Table S5.

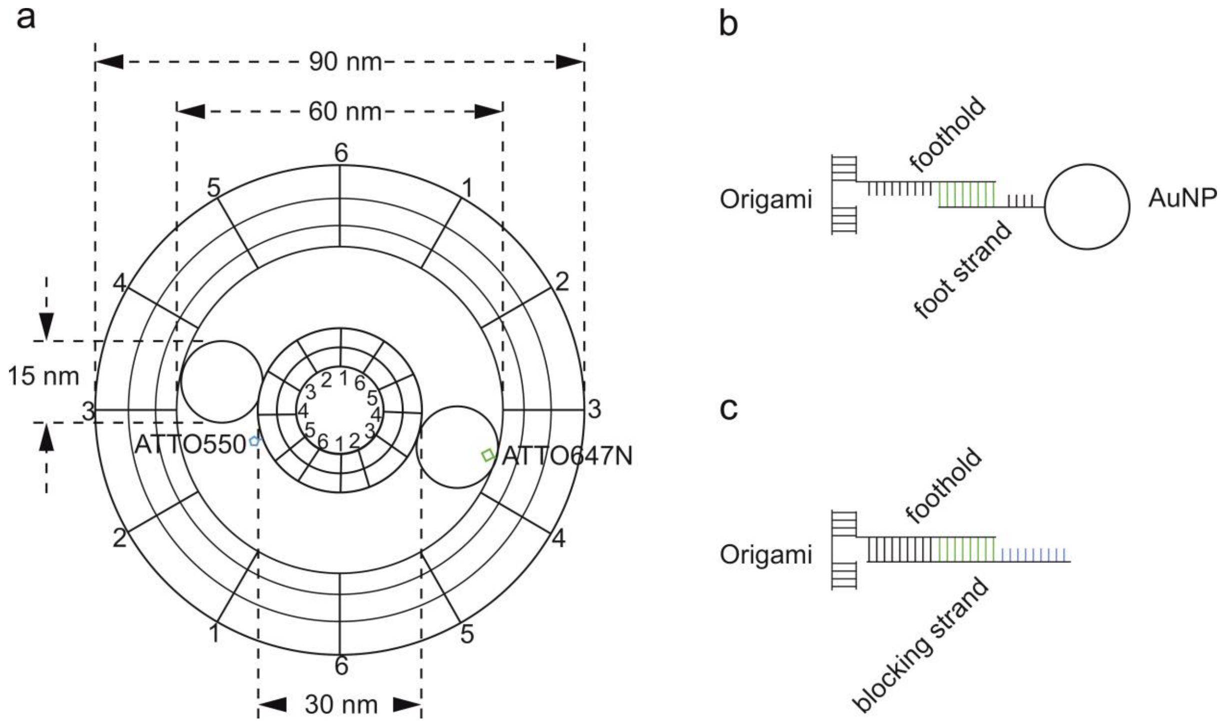

Supplementary Figure S5. (a) Schematic of the planetary gearset nanodevice. The outer and inner diameters of the large ring are ~90 nm and ~60 nm, respectively. The outer diameter of the small ring is ~30 nm. The AuNP diameter is 15 nm. (b) Schematic of the foothold-AuNP interaction. (c) Foothold design and blocking/releasing scheme. Each foothold comprises a unique stem region (black) connected to the DNA origami structure and a AuNP-capture sequence (green) to bind the AuNPs. Blocking strand deactivates the foothold by hybridizing with the stem and AuNP-capturing regions. Each blocking strand further comprises a toehold region (blue). Activation of the foothold is achieved by the addition of the complementary releasing strand, which completely hybridizes with the blocking strand to release it from the foothold.

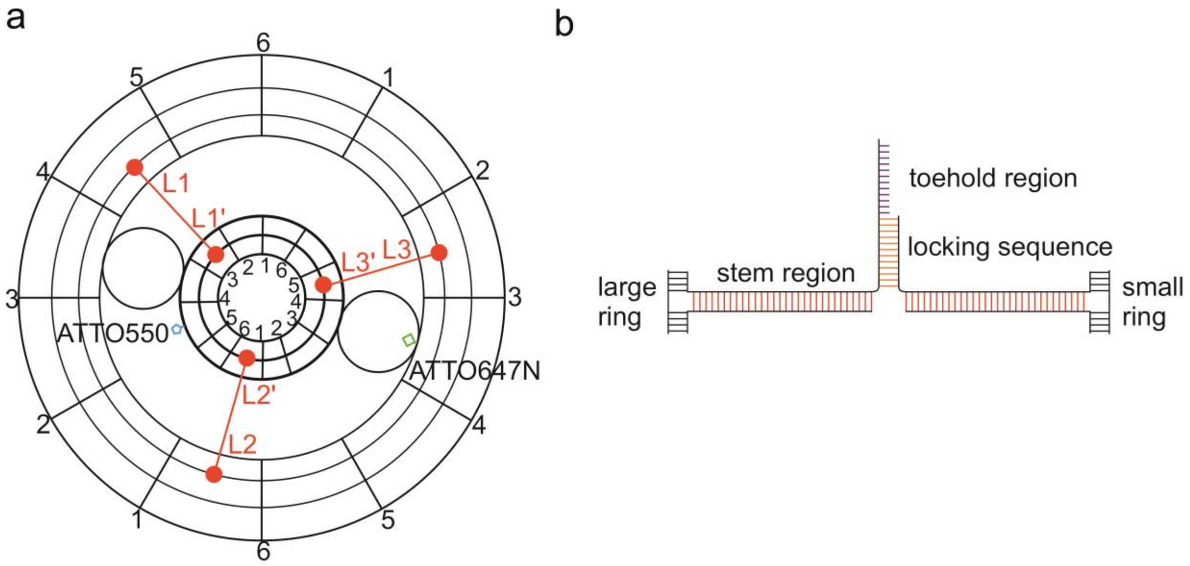

Supplementary Figure S6. (a) Schematic of the rotary nanodevice indicated with locking positions. Three orthogonal, complementary locking couples (L1-L1’, L2-L2’ and L3-L3’, red) are placed onto the large and small DNA origami rings to direct the orientation and relative position of the rings. (b) Design of the locking strands. Each lock comprises a stem region (red) and a locking sequence (orange). The linker strand of the large ring further comprises a toehold region (purple) to enable the opening of the locking couple through toehold-mediated strand displacement reactions.

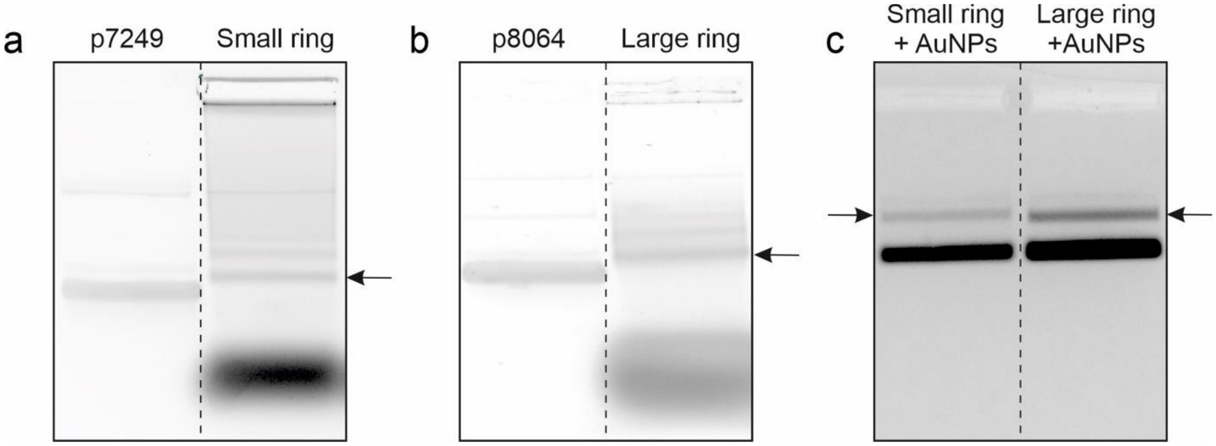

Supplementary Figure S7. (a) Agarose gel electrophoresis image of the small DNA origami ring after assembly. The band marked with the arrow contains the desired origami structures. The p7249 scaffold is used as reference. (b) Agarose gel electrophoresis images of the large DNA origami ring after assembly. The band marked with the arrow contains the desired origami structures. The p8064 scaffold is used as reference. (c) Agarose gel electrophoresis image of the AuNP-functionalized small and large origami rings after the annealing process. The bands marked with the arrows contain the desired nanostructures. The intense black bands contain excess AuNPs.

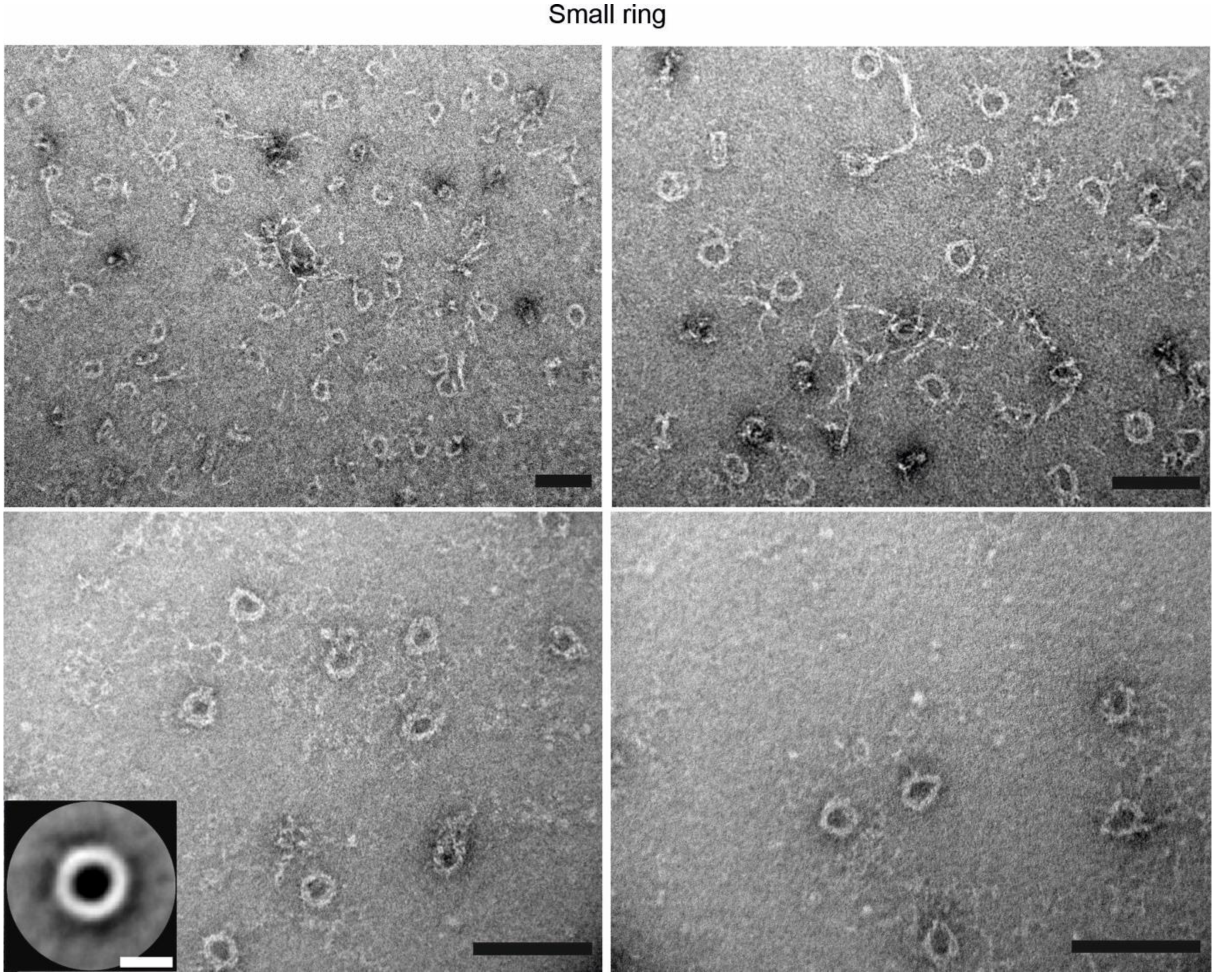

Supplementary Figure S8. TEM images of the small origami rings. Scale bar, 100 nm. Inset: averaged TEM image (see Supplementary Figure S9 for the structure library used for the calculation). Scale bar, 20 nm.

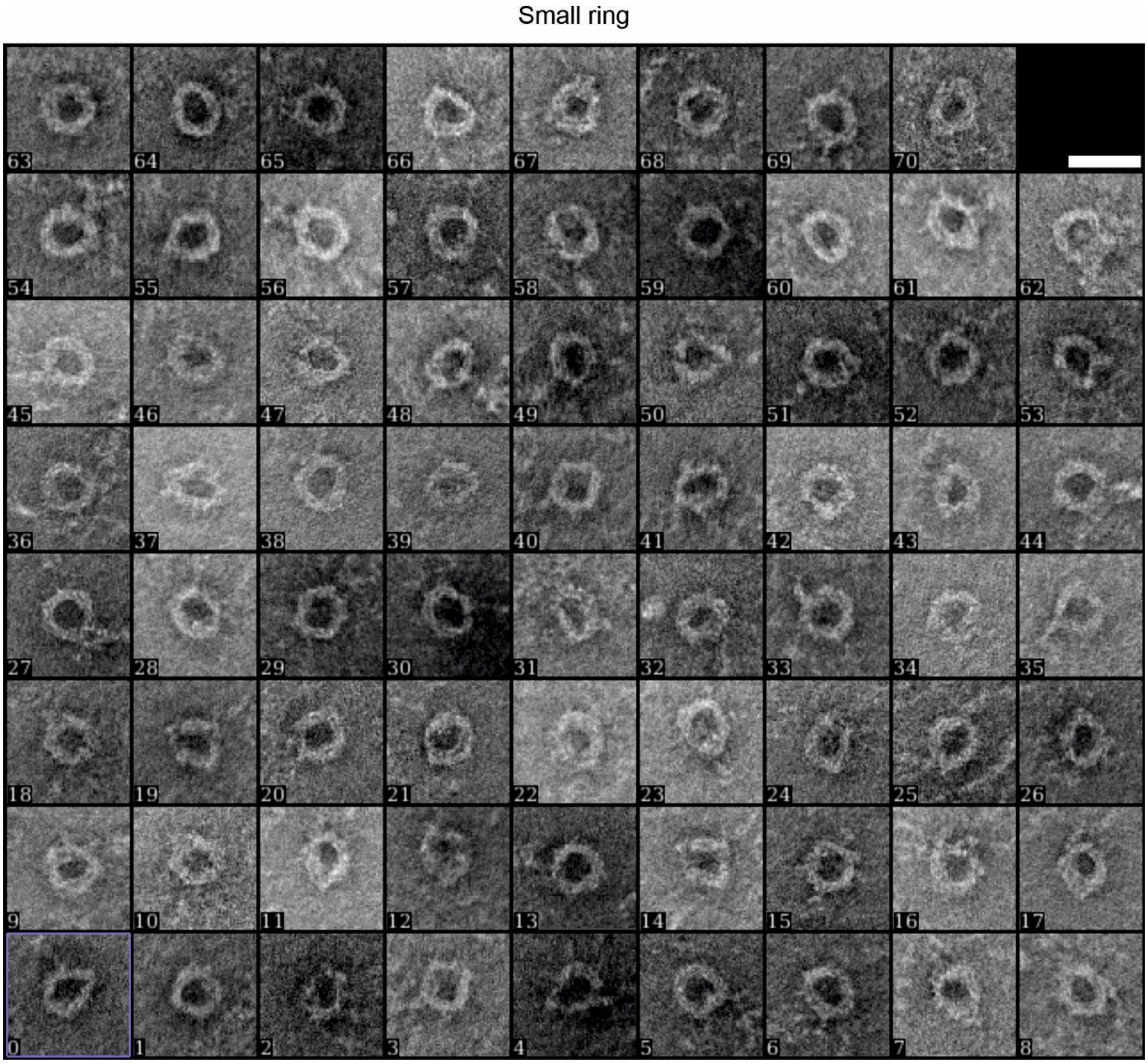

Supplementary Figure S9. Structure library of the small origami rings to calculate the averaged TEM image (see Supplementary Figure S8). Scale bar, 50 nm.

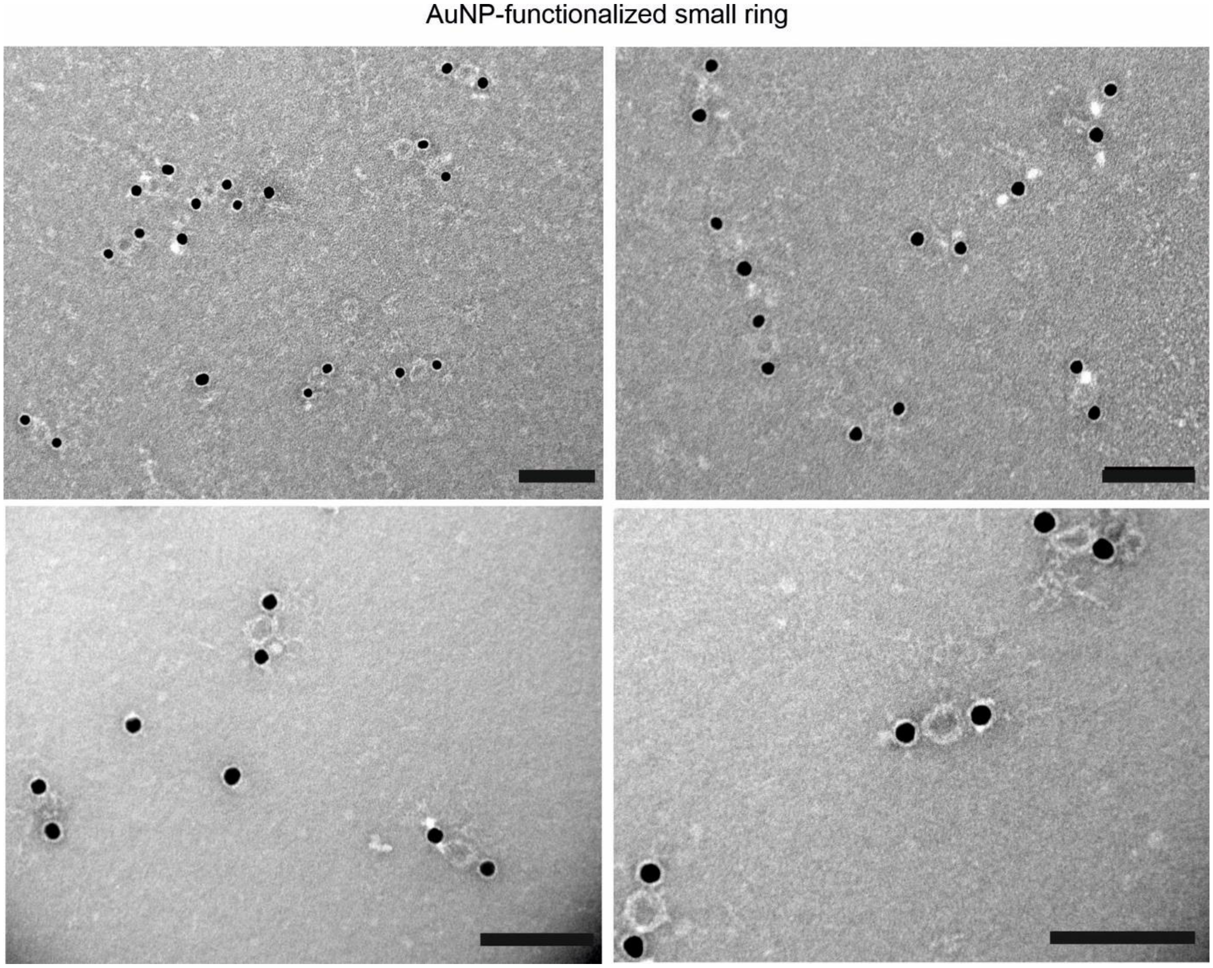

Supplementary Figure S10. TEM images of the AuNP-functionalized small origami rings. Scale bar, 100 nm.

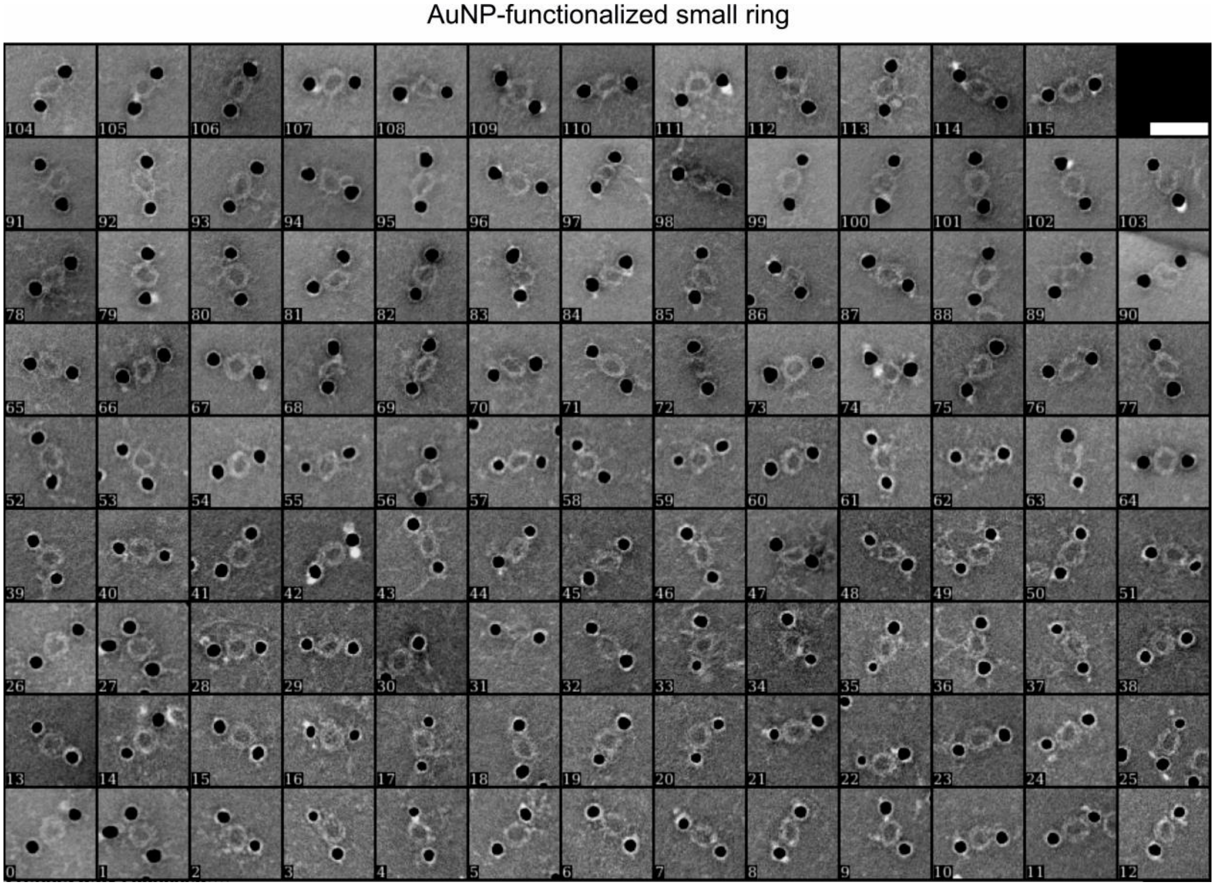

Supplementary Figure S11. Structure library of the AuNP-functionalized small origami rings to calculate the averaged TEM image (see Figure 2b). Scale bar, 50 nm.

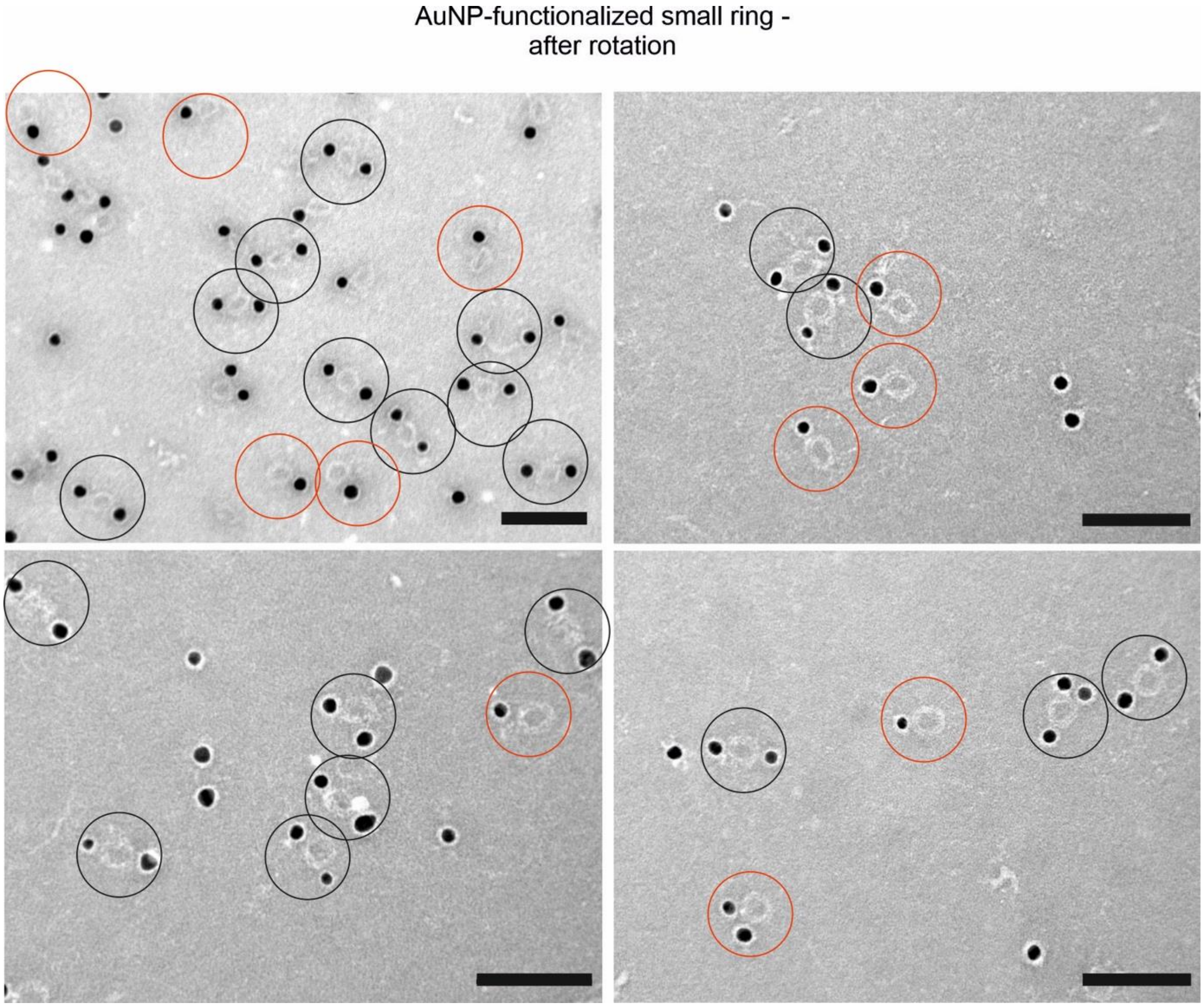

Supplementary Figure S12. TEM images of the AuNP-functionalized small origami rings after the rotation process and classification of the structures. The structures are divided into intact (black) and defective (red) ones. Some structures might have been defective directly after the structural assembly, but not from the rotation process. Scale bar, 100 nm.

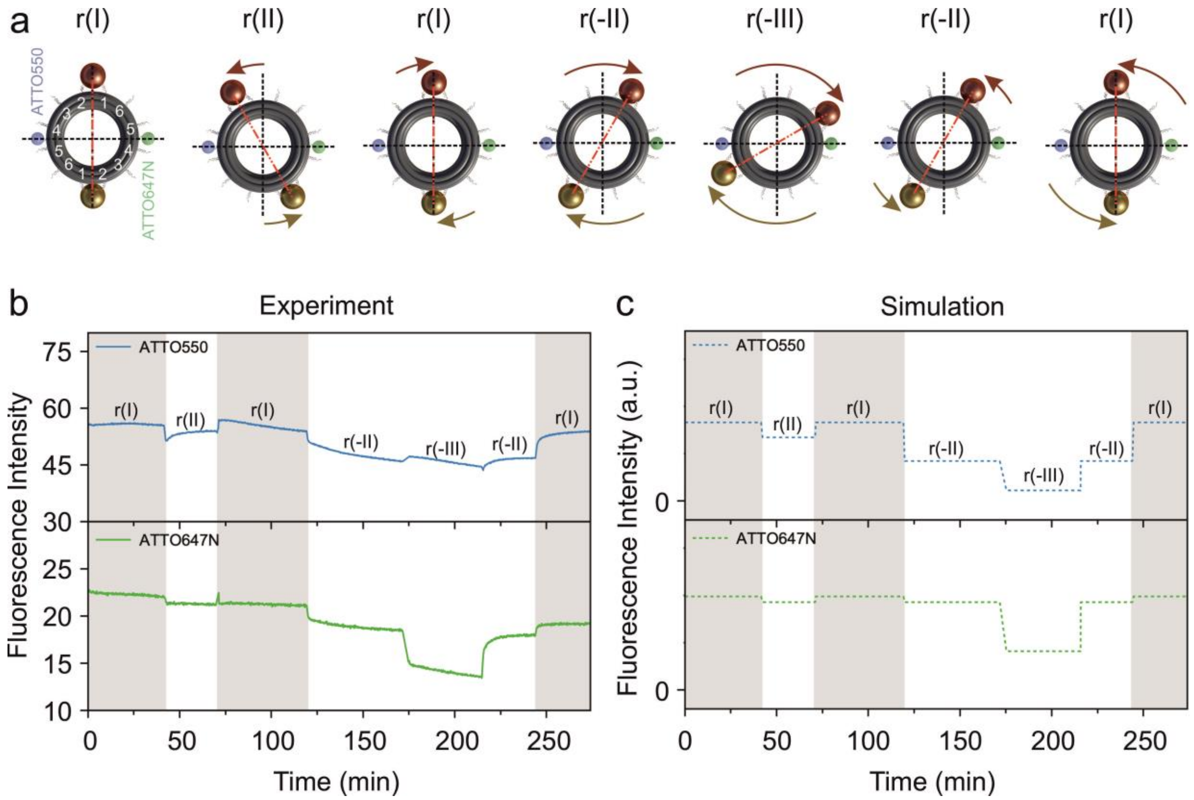

Supplementary Figure S13. (a) Bidirectional rolling of the AuNPs along the small origami ring (for exact angle changes see Supplementary Figure S4). (b) Experimental fluorescence data. (c) Calculated fluorescence data.

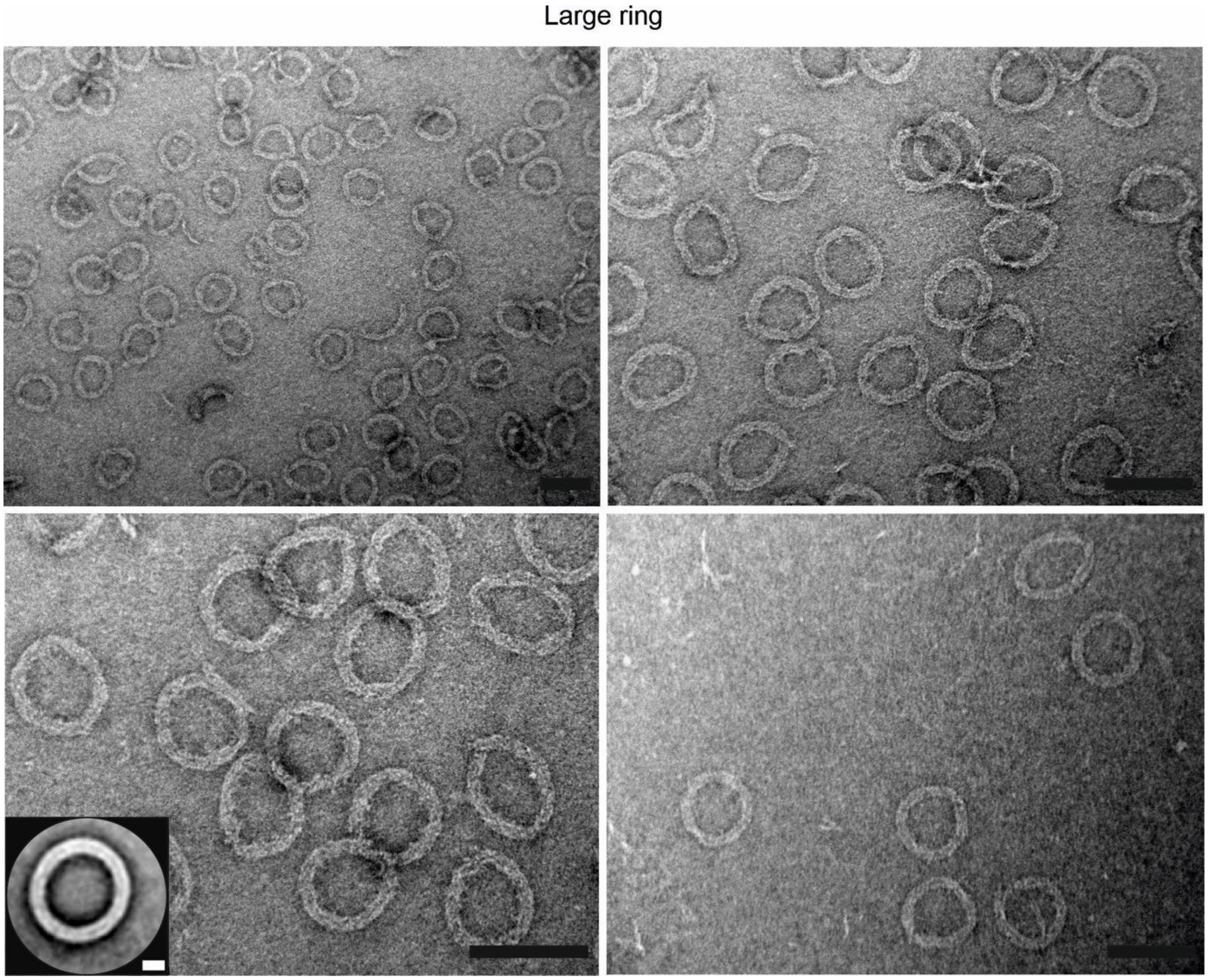

Supplementary Figure S14. TEM images of the large origami rings. Scale bar, 100 nm. Inset: averaged TEM image (see Supplementary Figure S14 for the structure library used for the calculation). Scale bar, 20 nm.

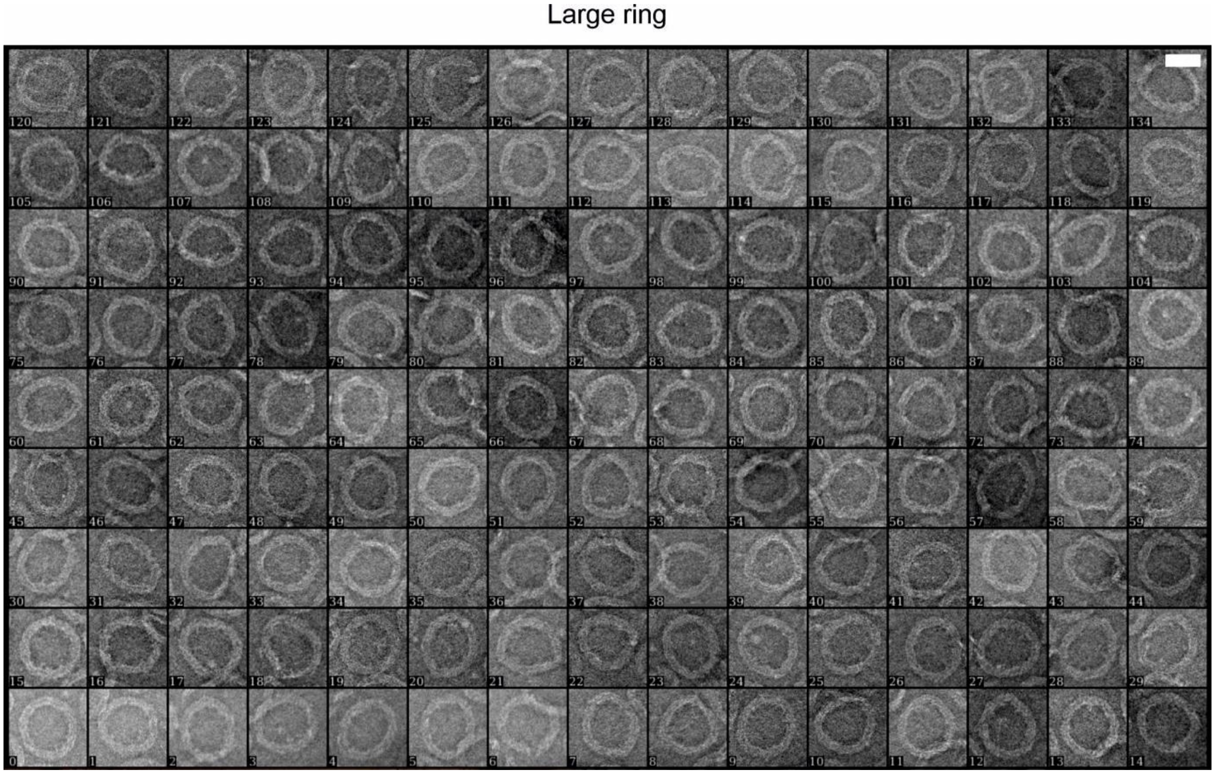

Supplementary Figure S15. Structure library of the large origami rings to calculate the averaged TEM image (see Supplementary Figure S13). Scale bar, 50 nm.

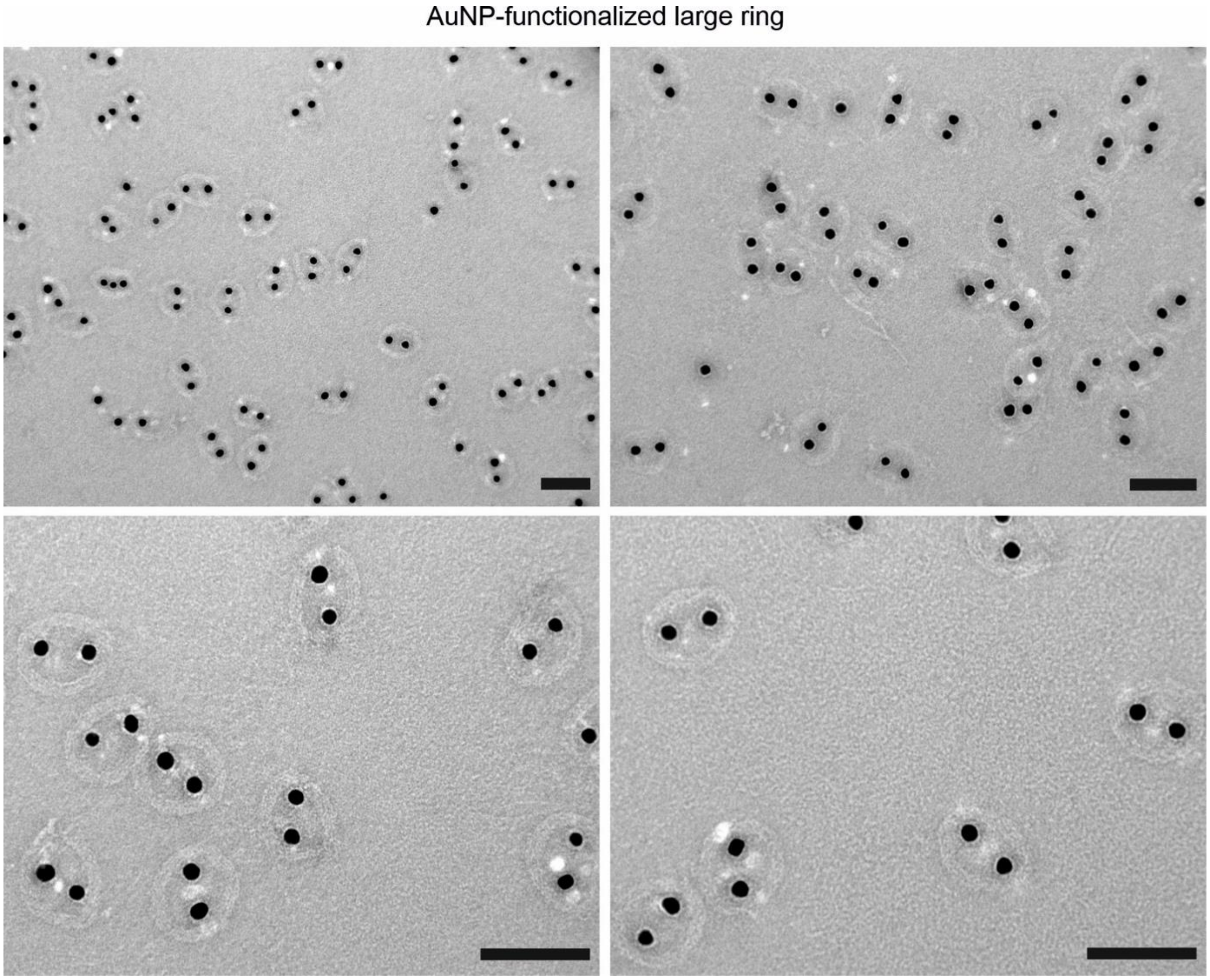

Supplementary Figure S16. TEM images of the AuNP-functionalized large origami rings. Scale bar, 100 nm.

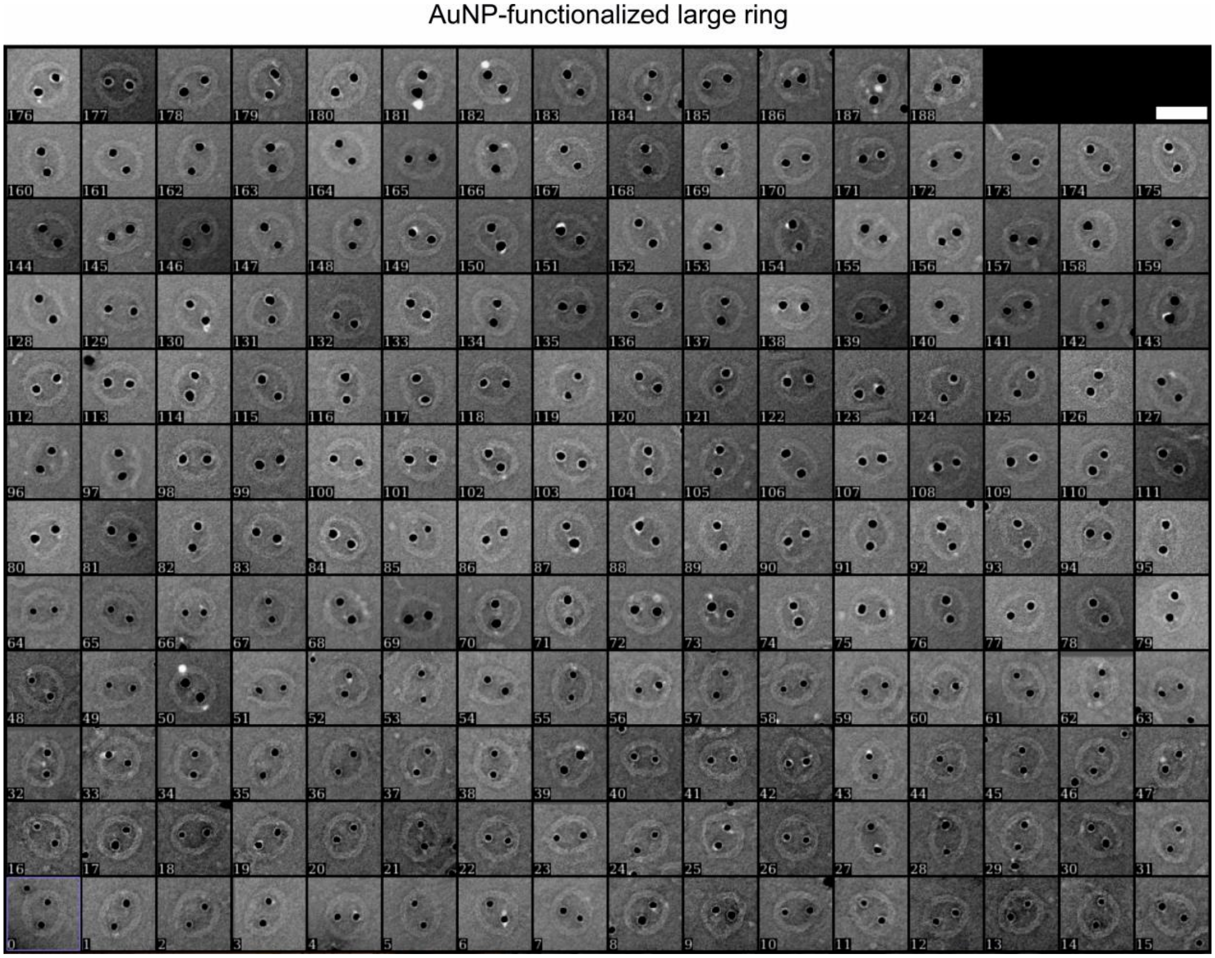

Supplementary Figure S17. Structure library of the AuNP-functionalized large origami rings to calculate the averaged TEM image (see Figure 3b). Scale bar, 100 nm.

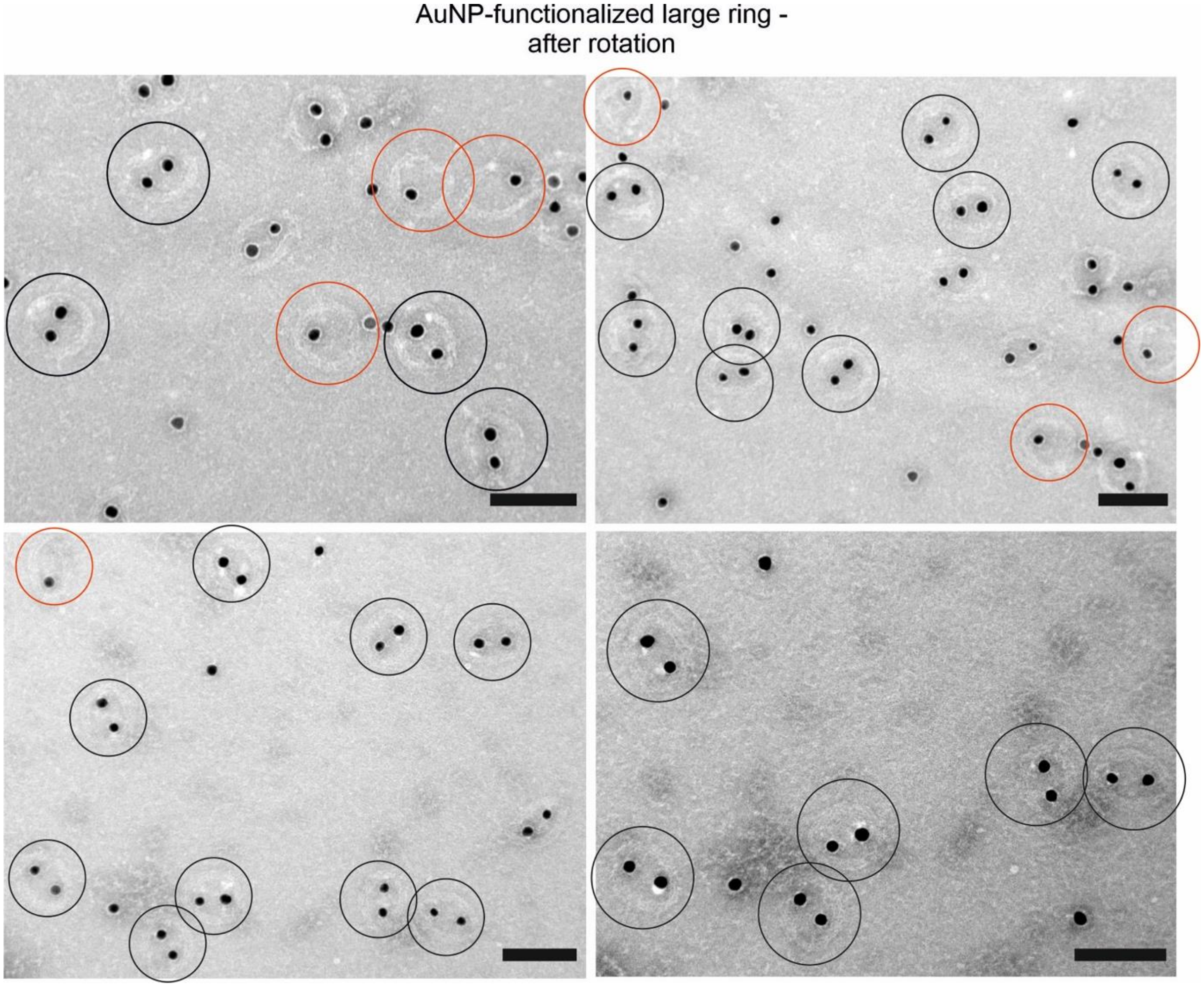

Supplementary Figure S18. TEM images of the AuNP-functionalized large origami rings after the rotation process and classification of the structures. The structures are divided into intact (black) and defective (red) ones. Some structures might have been defective directly after the structural assembly, but not from the rotation process. Scale bar, 100 nm.

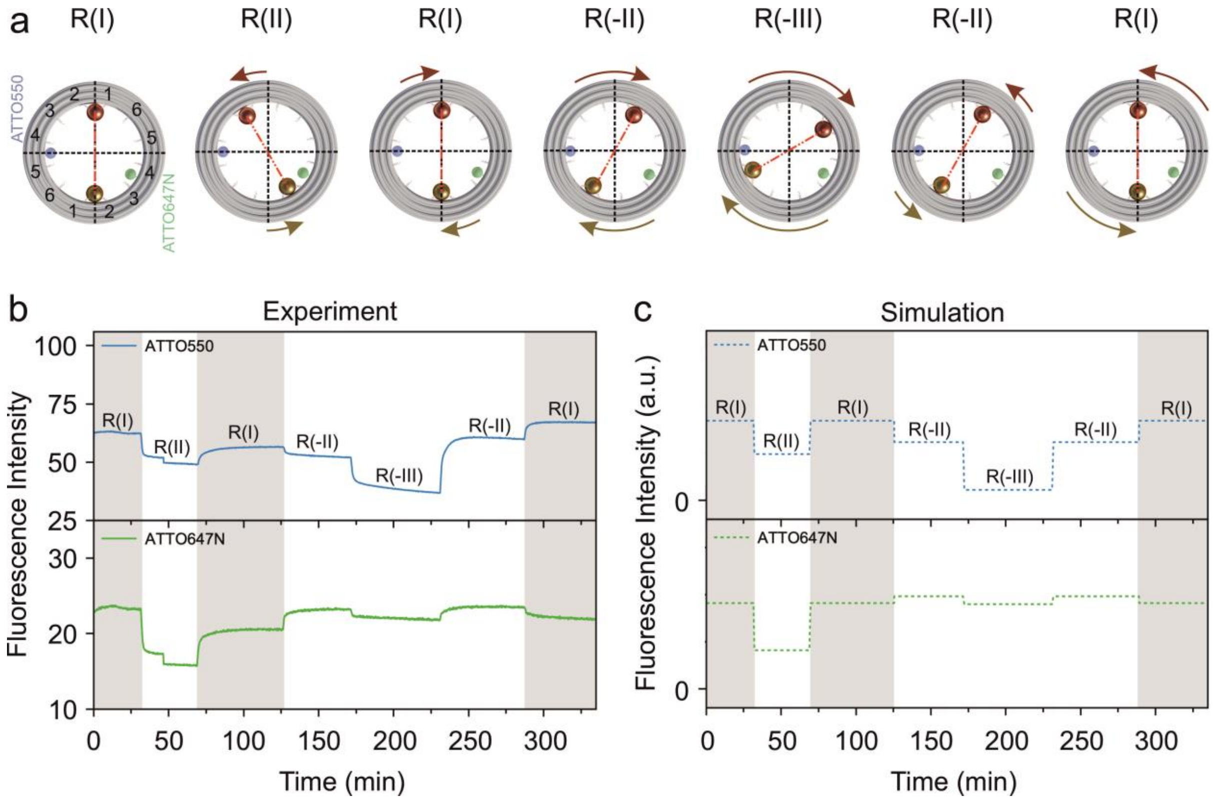

Supplementary Figure S19. (a) Bidirectional rolling of the AuNPs along the large origami ring (for exact angle changes see Supplementary Figure S3). (b) Experimental fluorescence data. (c) Calculated fluorescence data.

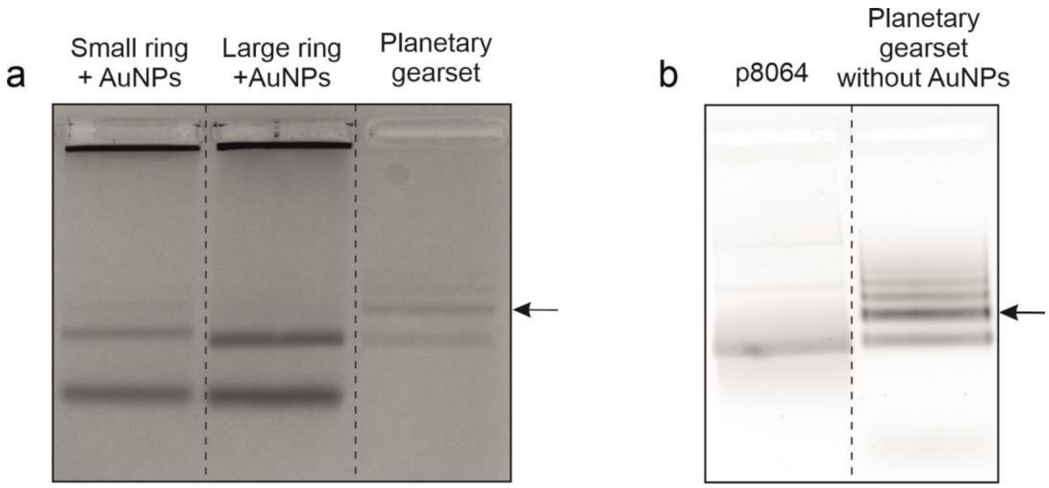

Supplementary Figure S20. (a) Agarose gel electrophoresis image of the assembled rotary nanodevices. The AuNP-functionalized small and large origami rings are used as reference. The band marked with the arrow contains the desired nanostructures. (b) Agarose gel electrophoresis images of the small and large origami rings connected only by the locking strands. The band marked with the arrow contains the desired double ring structures. The p8064 scaffold is used as reference.

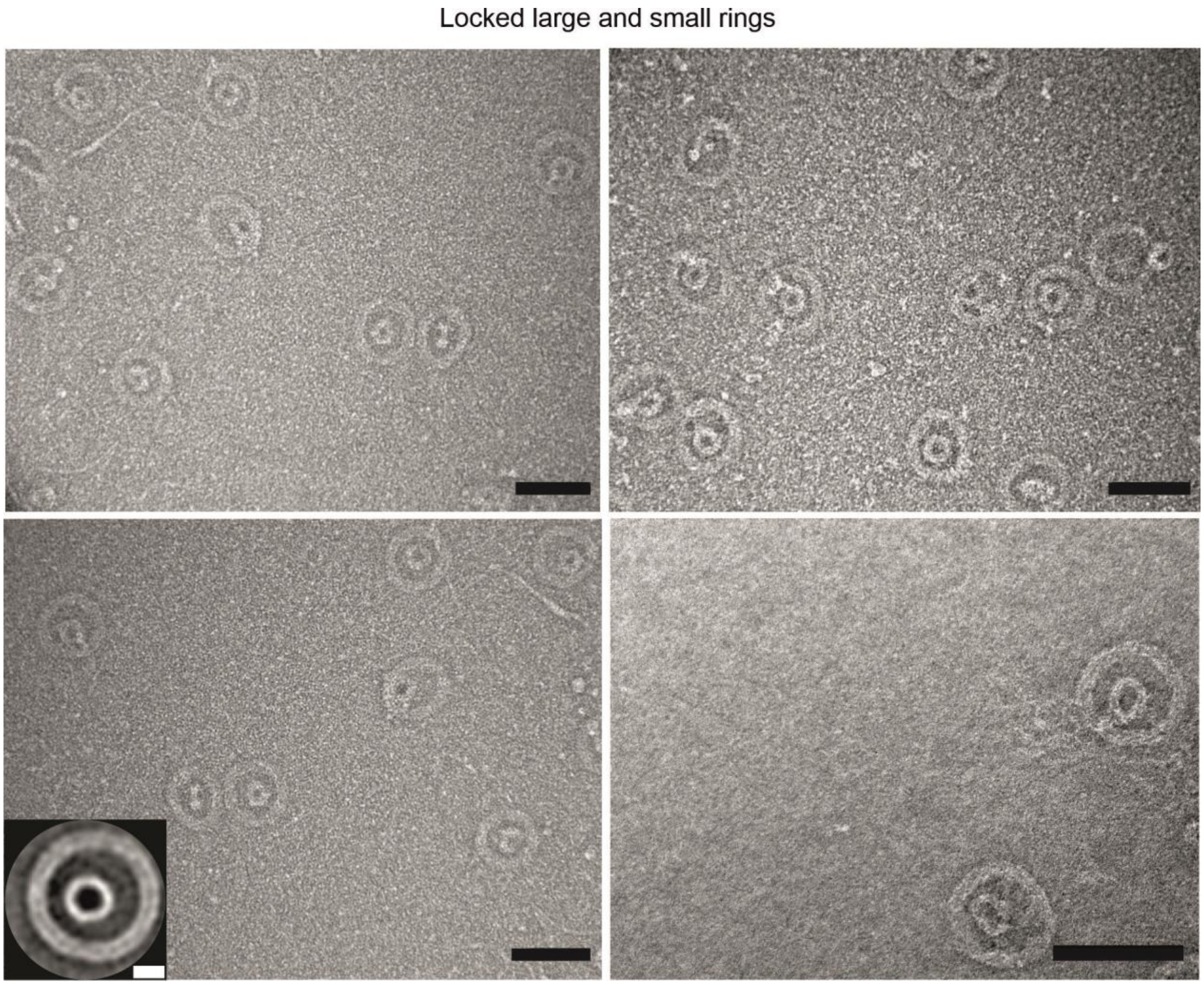

Supplementary Figure S21. TEM images of the double rings connected by the locking strands only. Scale bar, 100 nm. Inset: averaged TEM image (see Supplementary Figure S20 for the structure library used for the calculation). Scale bar, 20 nm.

Locked large and small rings

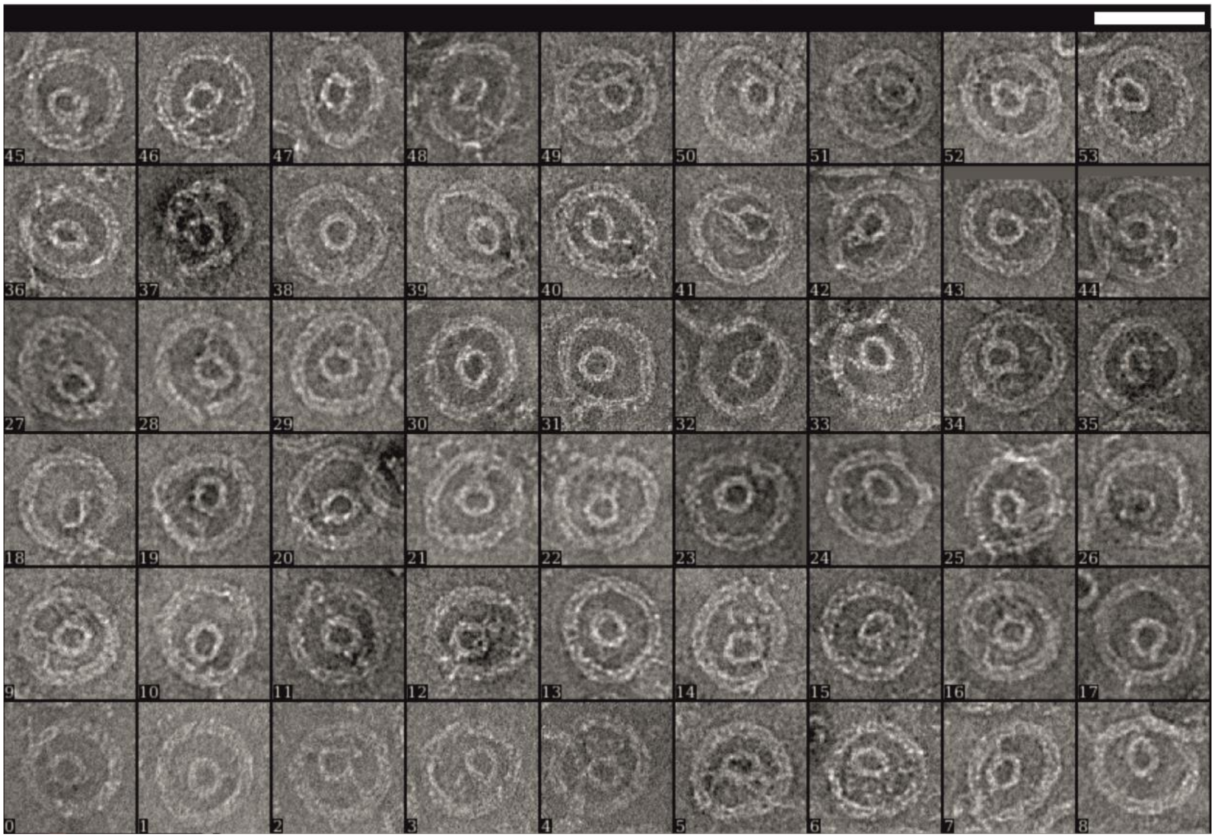

Supplementary Figure S22. Structure library of the connected double rings to calculate the averaged TEM image (see Supplementary Figure S19). Scale bar, 100 nm.

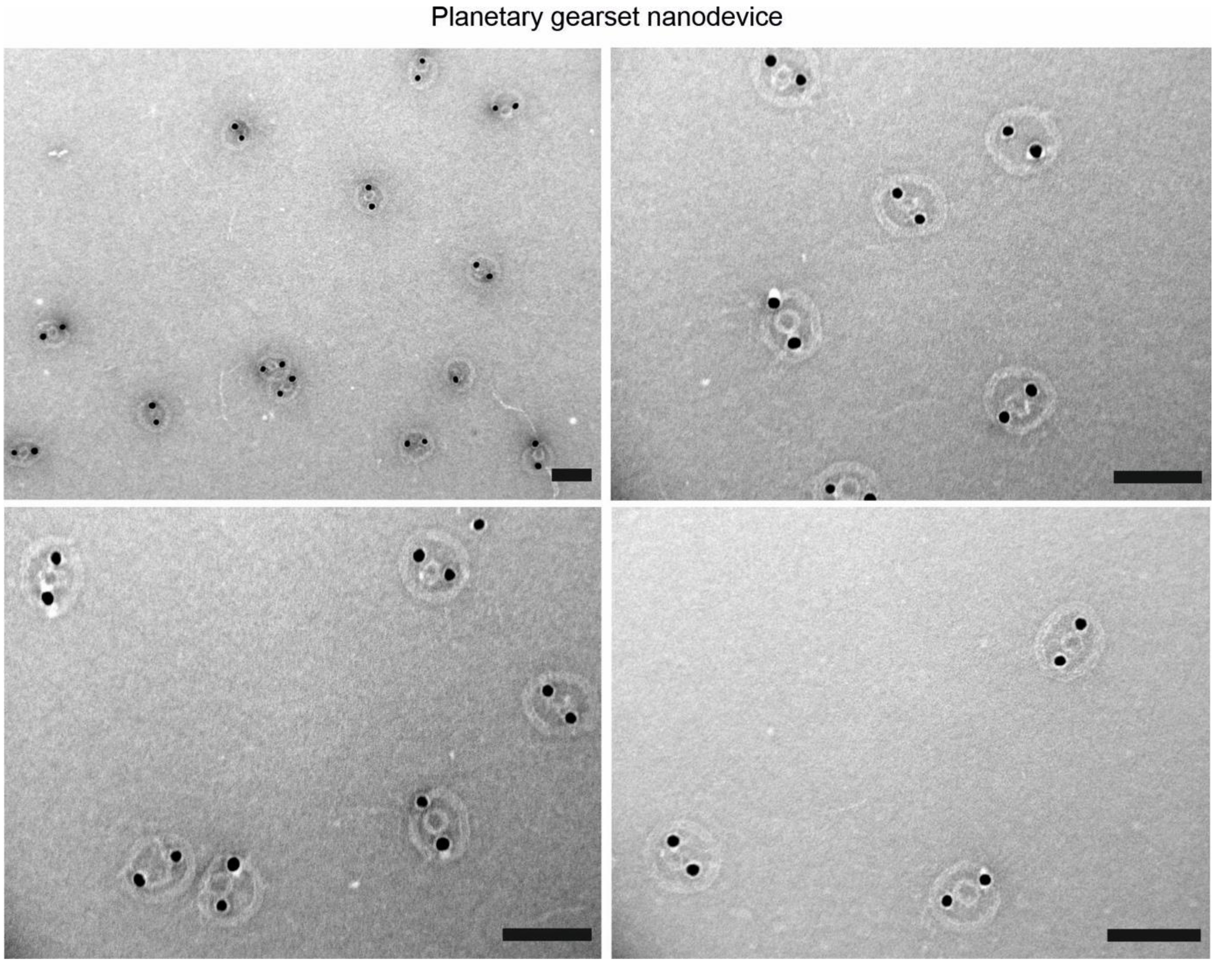

Supplementary Figure S23. TEM images of the assembled planetary gearset nanodevices. Scale bar, 100 nm.

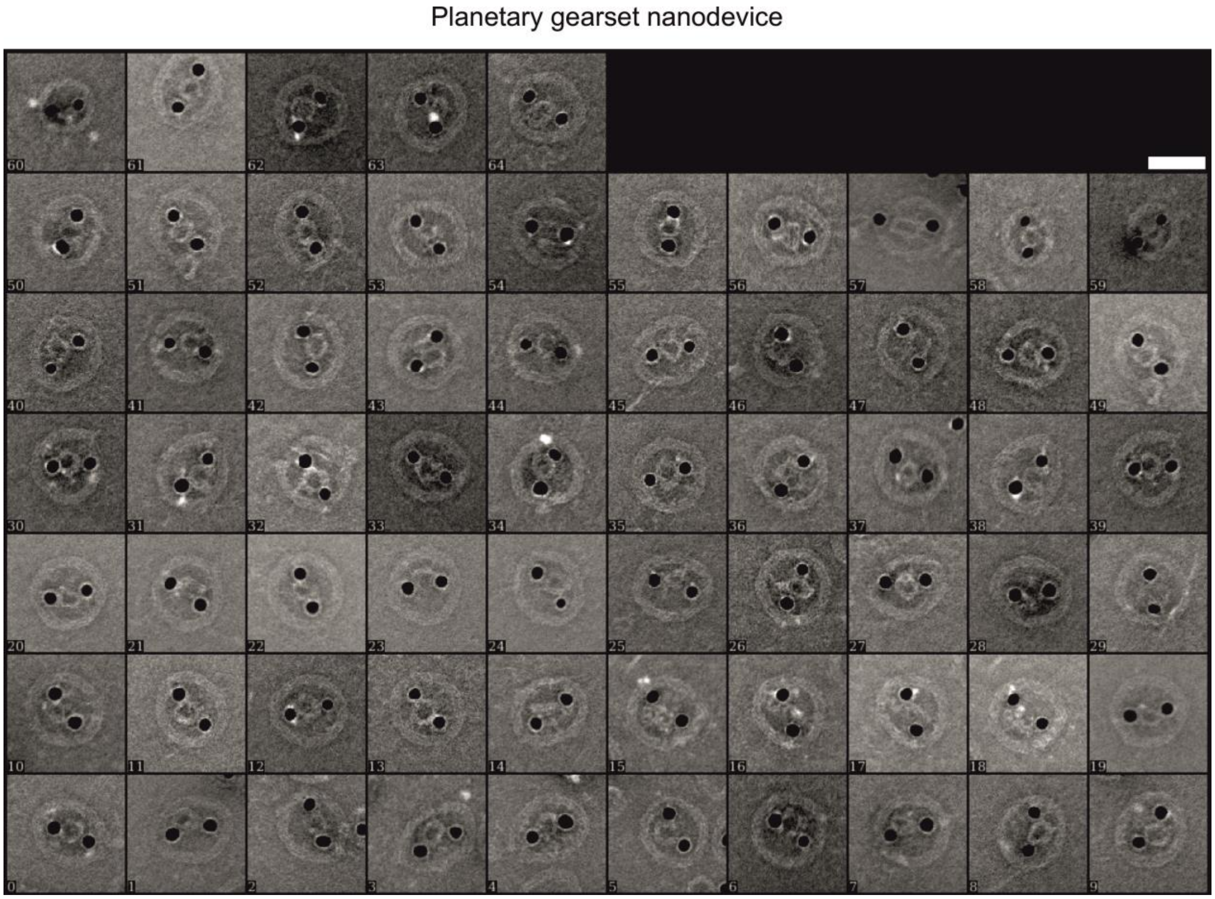

Supplementary Figure S24. Structure library of the planetary gearset nanodevices to calculate the averaged TEM image (see Figure 4b). Scale bar, 100 nm.

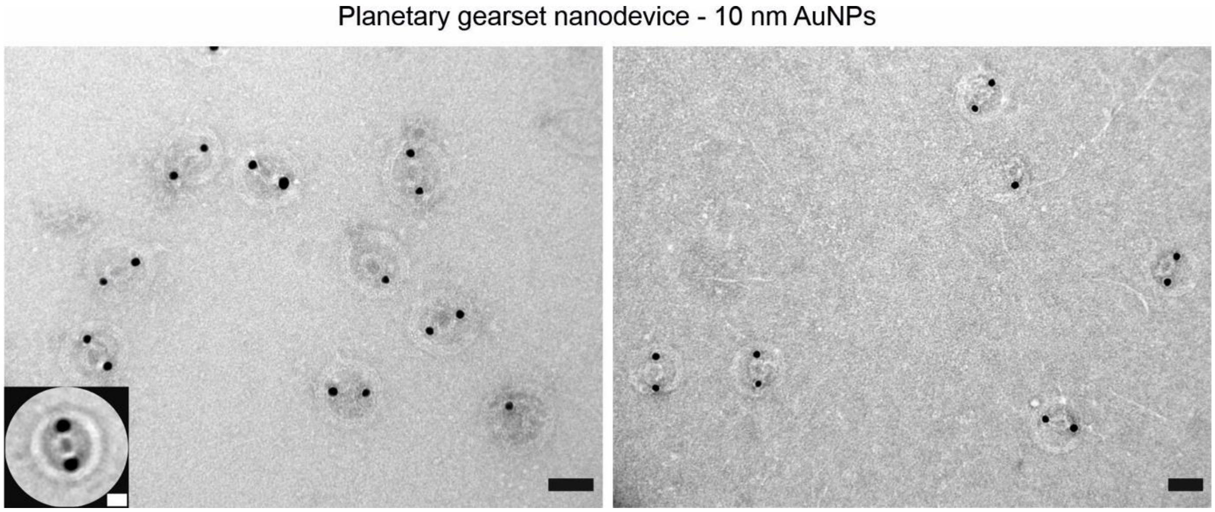

Supplementary Figure S25. TEM images of the planetary gearsets with 10 nm AuNPs. Scale bar, 100 nm. Inset: averaged TEM image (see Supplementary Figure S26 for the structure library used to calculate the averaged TEM image). Scale bar, 20 nm. Due to the less tightly-fitted geometry, the small rings show weaker deformations on the TEM grid after the deposition and drying processes.

Planetary gearset nanodevice with 10 nm AuNPs

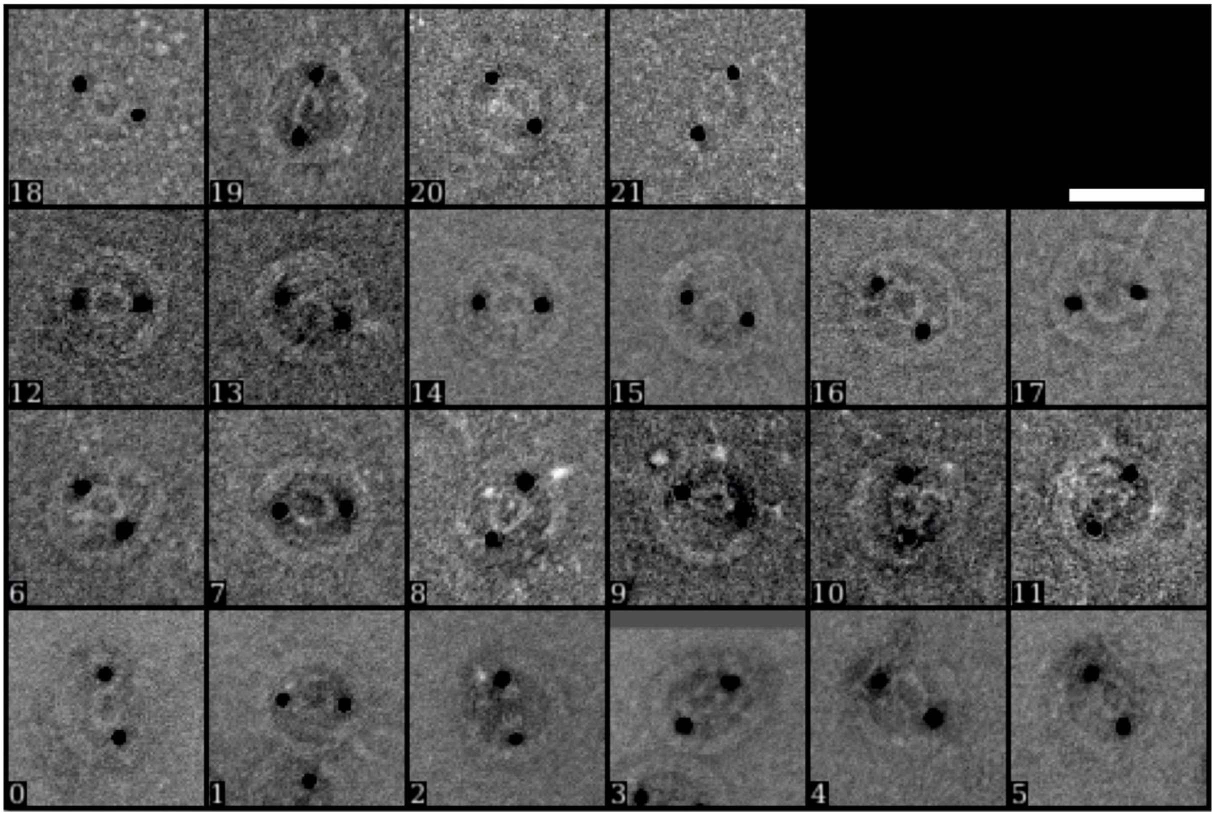

Supplementary Figure S26. Structure library of the planetary gearsets with 10 nm AuNPs to calculate the averaged TEM image. Scale bar, 100 nm.

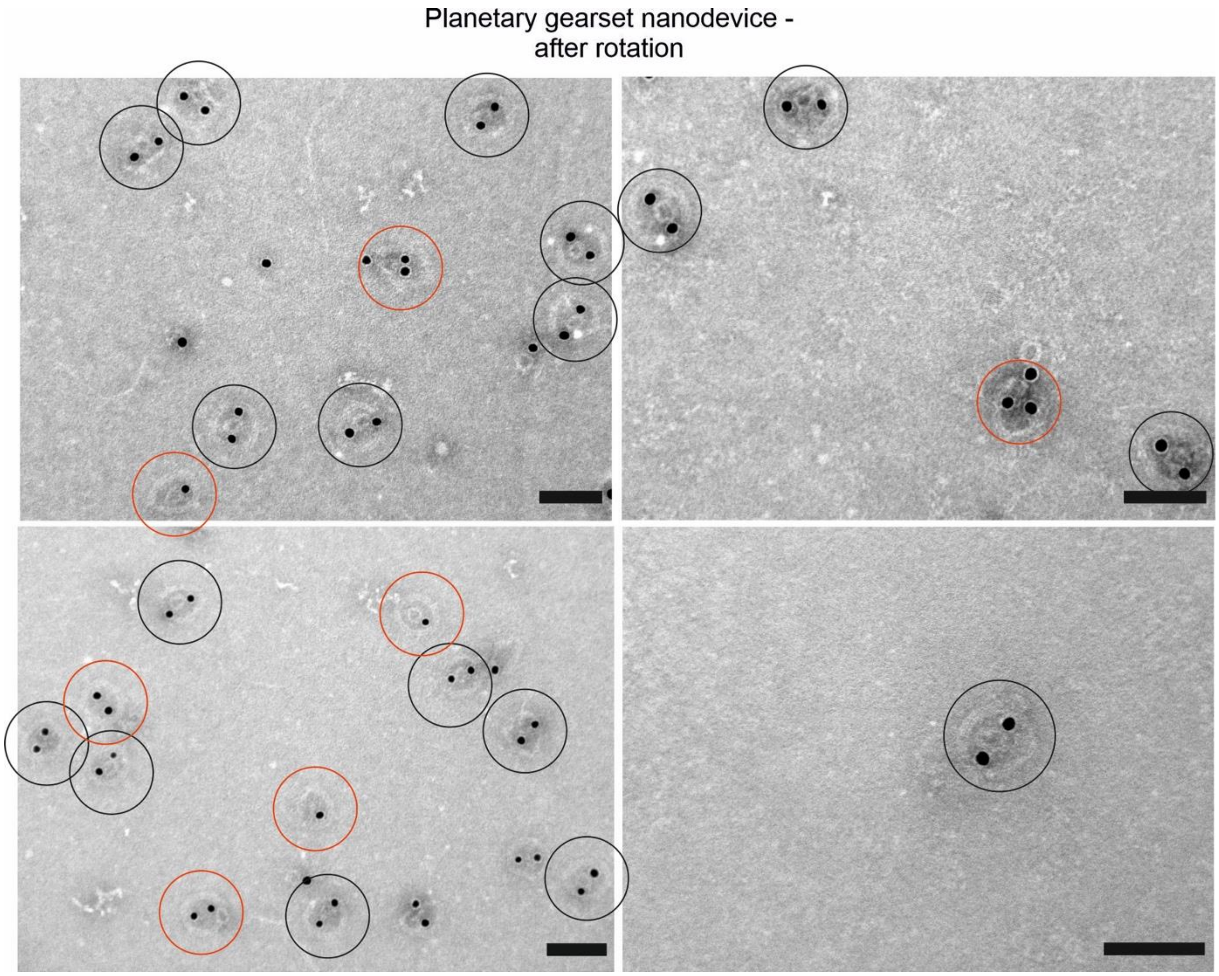

Supplementary Figure S27. TEM images of the rotary nanodevices after the rotation process and classification of the structures. The structures are divided into intact (black) and defective (red) ones. Note that some of the defective structures might have been formed directly after the structural assembly but not be due to the rotation process. Scale bar, 100 nm.

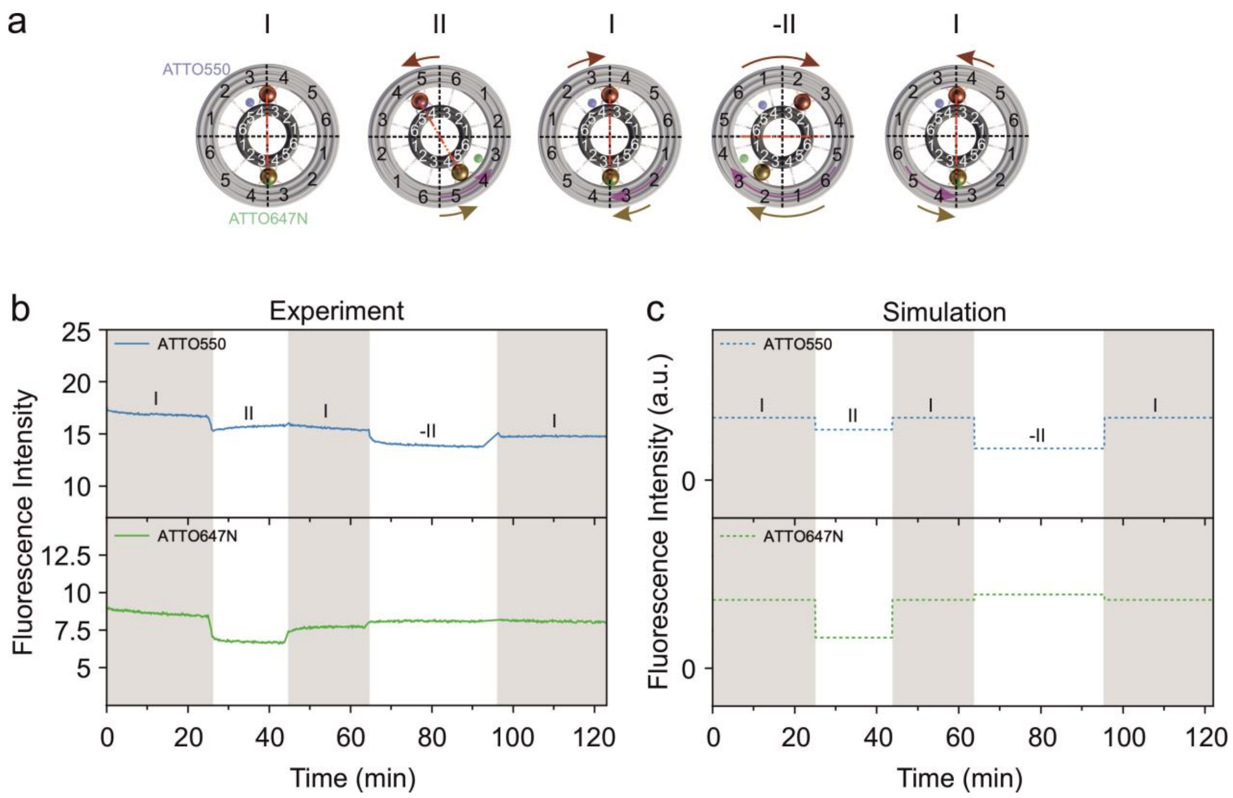

Supplementary Figure S28. (a) Bidirectional sliding of the AuNPs in between the small and large origami rings (for exact angle changes see Supplementary Figures S3 and S4). (b) Experimental fluorescence data. (c) Calculated fluorescence data.

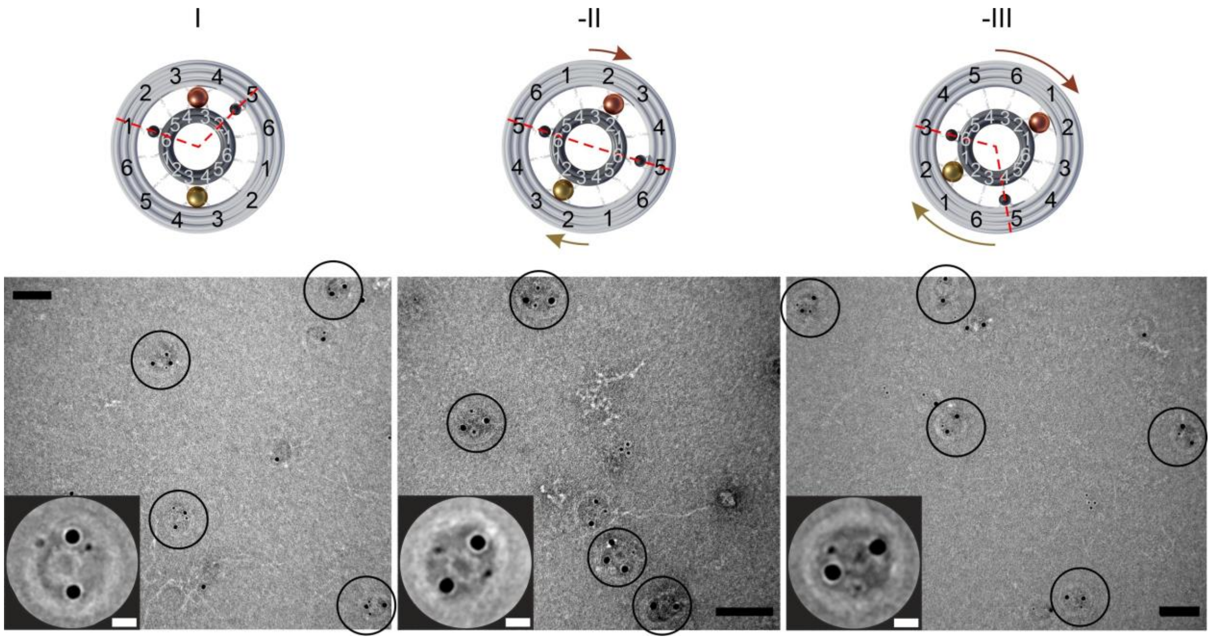

Supplementary Figure S29. Additional AuNPs as markers to validate the rotation mechanism. One AuNP is immobilized at foothold row 6 on the small ring, while the other AuNP is positioned at foothold row 5 on the large ring. Schematic of three representative states 'I', '-II', and '-III', in which the two new AuNPs (5 nm) are illustrated using black balls. The gearset is driven from state 'I' to '-III' and the corresponding TEM images at these states clearly prove the designated relative movements among the AuNPs, which agree well with the predictions based on our rotation mechanism. Scale bar, 100 nm. Inset: averaged TEM images (see Supplementary Figures S30 to S32 for the structure libraries used to calculate the averaged TEM images). Scale bar, 20 nm.

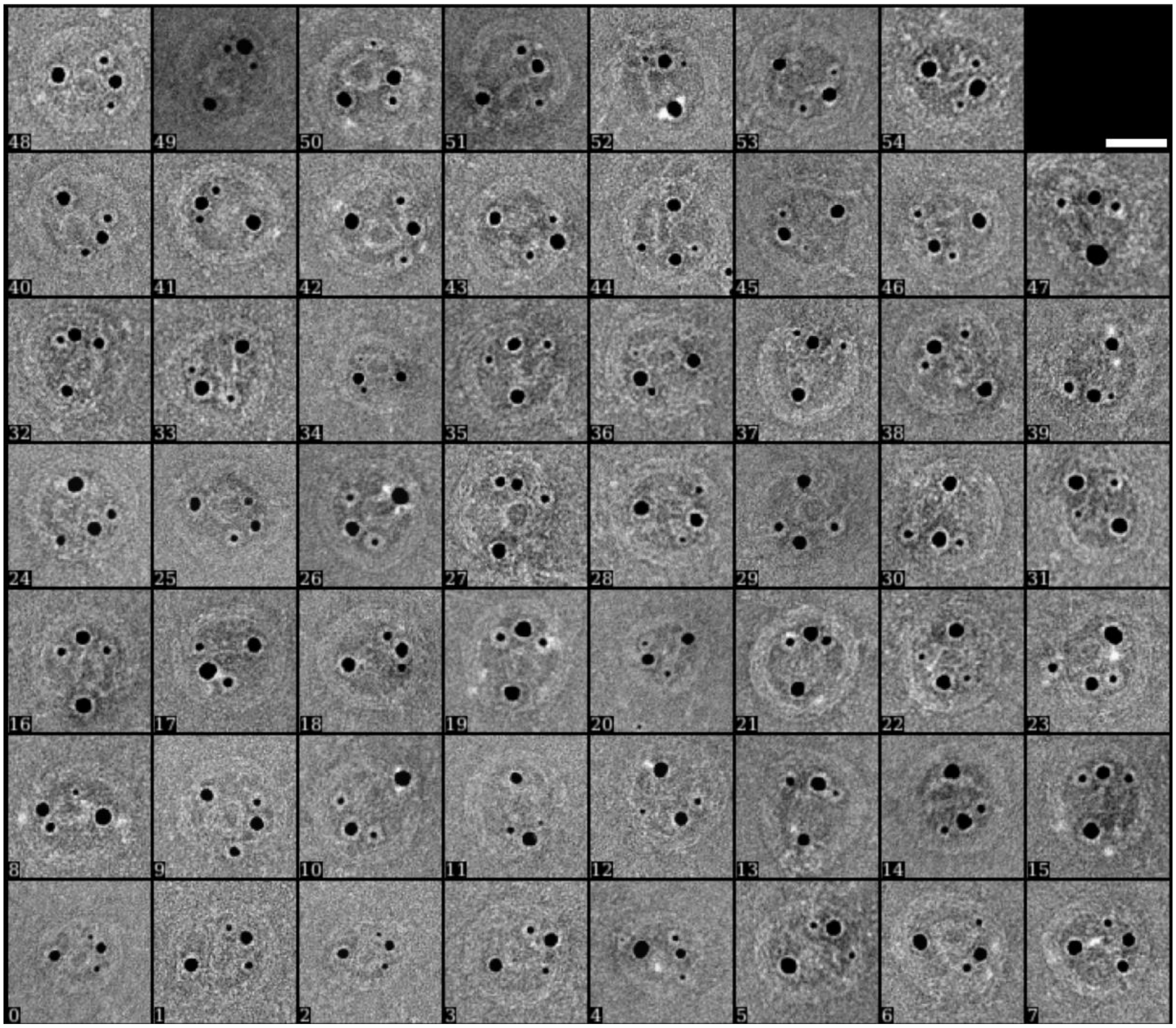

Supplementary Figure S30. Structure library of the planetary gearset nanodevices at state 'I' to calculate the averaged TEM image (see Supplementary Figure 29). Scale bar, 50 nm.

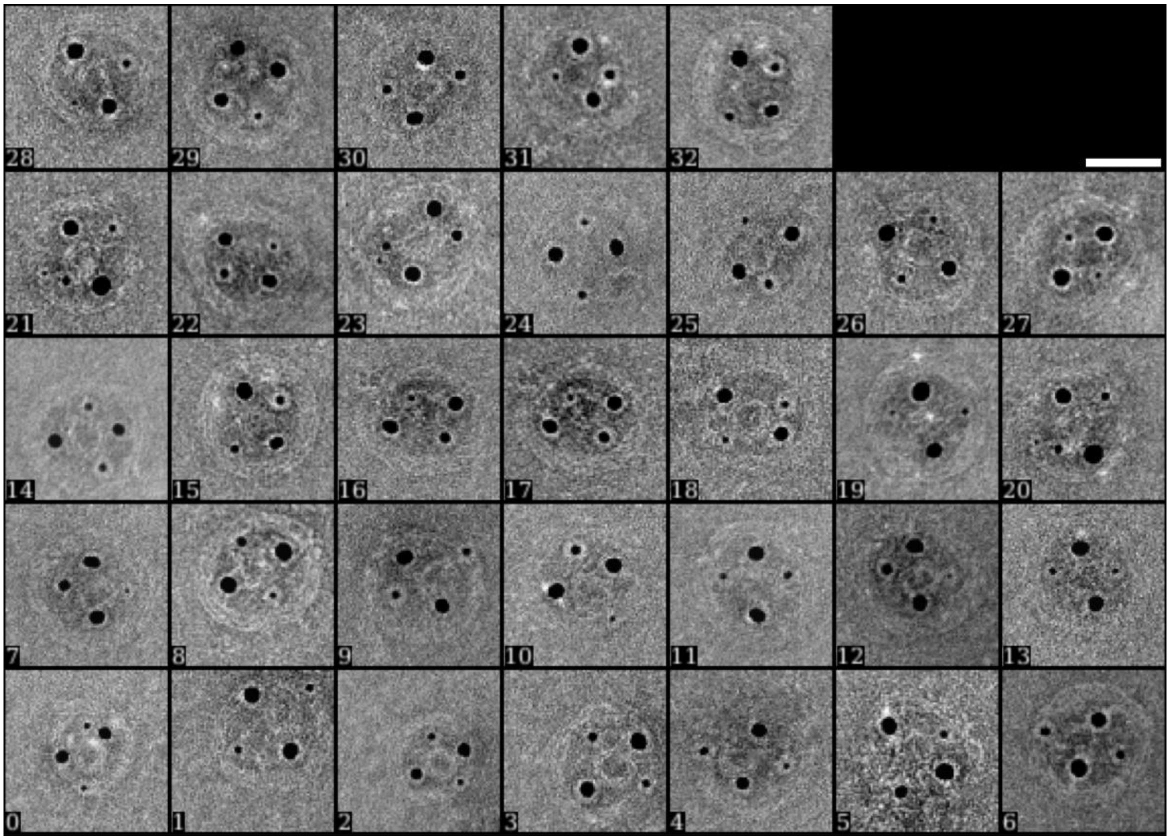

Supplementary Figure S31. Structure library of the planetary gearset nanodevices at state '-II' to calculate the averaged TEM image (see Supplementary Figure 29). Scale bar, 50 nm.

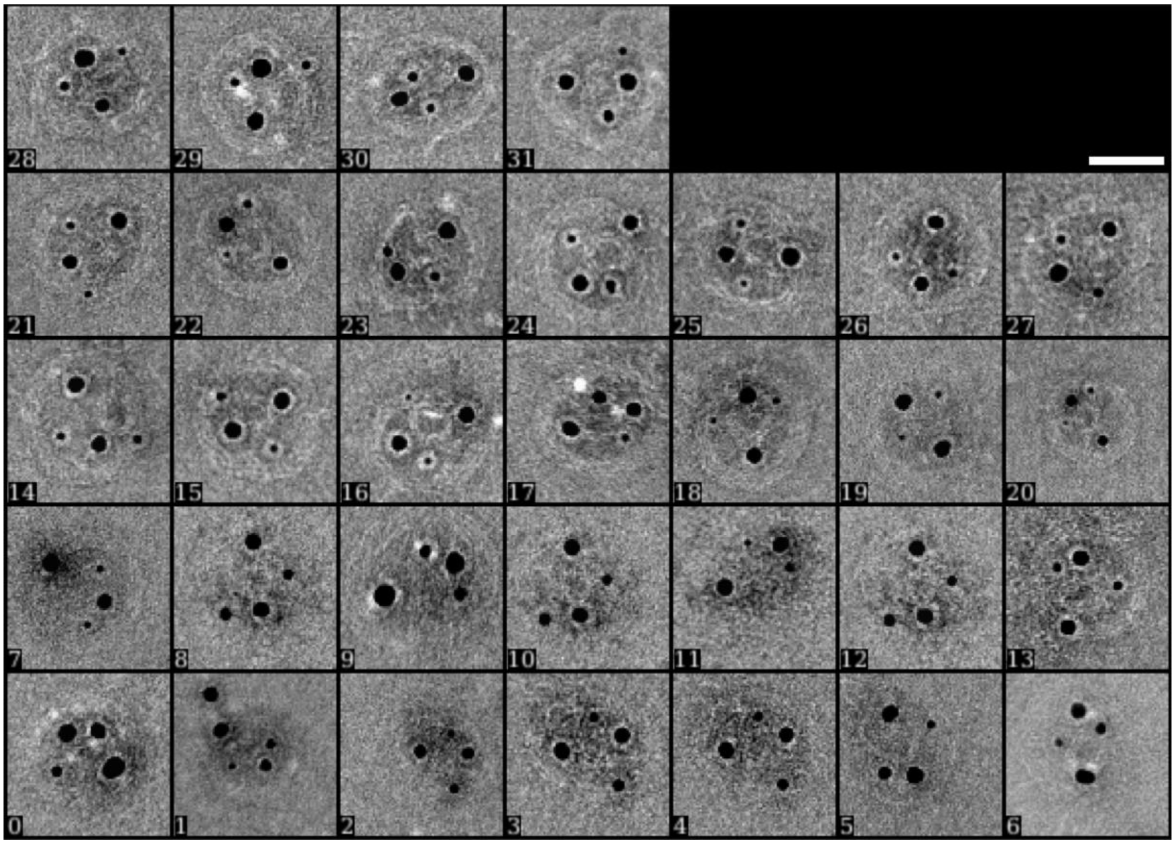

Supplementary Figure S32. Structure library of the planetary gearset nanodevices at state '-III' to calculate the averaged TEM image (see Supplementary Figure 29). Scale bar, 50 nm.

Supplementary Tables

Supplementary Table S1. Samples added to drive the rotation for real time fluorescence detection for the small rings in Figure 2. The blocking and releasing strands were prepared at a concentration of 400 µM in H2O. Generally, small volumes but high concentrations of the blocking and releasing DNA strands were used to power the AuNPs to reduce the dilution effect. The total volume increase was 4.5 µL (4.3%) after the rotation process.

Step	Strands added	Volume added per strand
‘r(I)’ to ‘r(II)’	1, $\bar{3}$	0.25 µL, 0.25 µL
‘r(II)’ to ‘r(III)’	2, $\bar{4}$	0.25 µL, 0.25 µL
‘r(III)’ to ‘r(IV)’	3, $\bar{5}$	0.5 µL, 0.25 µL
‘r(IV)’ to ‘r(V)’	4, $\bar{6}$	0.5 µL, 0.25 µL
‘r(V)’ to ‘r(VI)’	5, $\bar{1}$	0.5 µL, 0.5 µL
‘r(VI)’ to ‘r(VII)’	6, $\bar{2}$	0.5 µL, 0.5 µL

Supplementary Table S2. Samples added to drive the rotation for real time fluorescence detection for the large rings in Fig. 3. The blocking and releasing strands were prepared at a concentration of 400 μM in H2O. To reduce the dilution effect, small volumes and high concentrations of the blocking and releasing DNA strands were used. After the rotation process, the total volume increase was 4.5 μL (4.3%).

Step	Strands added	Volume added per strand
'R(I)' to 'R(II)'	1, $\overline{3}$	0.25 μL, 0.25 μL
'R(II)' to 'R(III)'	2, $\overline{4}$	0.25 μL, 0.25 μL
'R(III)' to 'R(IV)'	3, $\overline{5}$	0.5 μL, 0.25 μL
'R(IV)' to 'R(V)'	4, $\overline{6}$	0.5 μL, 0.25 μL
'R(V)' to 'R(VI)'	5, $\overline{1}$	0.5 μL, 0.5 μL
'R(VI)' to 'R(VII)'	6, $\overline{2}$	0.5 μL, 0.5 μL

Supplementary Table S3. Samples added to drive the rotation for real time fluorescence detection for the planetary gearset devices in Fig. 4. The blocking and releasing strands were prepared at a concentration of 400 μM in H_2O. To reduce the dilution effect, small volumes and high concentrations of the blocking and releasing DNA strands were used. After the rotation process, the total volume increase was 4.2 μL (4%).

Step	Strands added	Volume added per strand
unlocking	Open locks L1, L2, L3	0.2 μL
'I' to 'II'	3, $\overline{5}$	0.2 μL, 0.4 μL
'II' to 'III'	4, $\overline{6}$	0.2 μL, 0.4 μL
'III' to 'IV'	5, $\overline{1}$	0.4 μL, 0.2 μL
'IV' to 'V'	6, 2	0.4 μL, 0.2 μL
'V' to 'VI'	1, $\overline{3}$	0.4 μL, 0.4 μL
'VI' to 'VII'	2, $\overline{4}$	0.4 μL, 0.4 μL

Supplementary Table S4. Distances between the fluorophores and the respective adjacent AuNPs used in the theoretical calculations for the large origami ring.

State	ATTO647N	ATTO550
	Distance to AuNP center (nm)	
'R(I)'	25	30.5
'R(II)'	16.5	23
'R(III)'	12.5	15.5
'R(IV)'	16	12.5
'R(V)'	24.5	16.5
'R(VI)'	32	25
'R(VII)'	25	30,5

Supplementary Table S5. Distances between the fluorophores and the respective adjacent AuNPs used in the theoretical calculations for the small origami ring.

State	ATTO647N	ATTO550
	Distance to AuNP center (nm)	
'r(I)'	34	30
'r(II)'	26	26
'r(III)'	16.5	16
'r(IV)'	12,5	12.5
'r(V)'	16.5	16.5
'r(VI)'	26	22
'r(VII)'	34	30

Supplementary Table S6. Distances between the fluorophores and the respective adjacent AuNPs used in the theoretical calculations for the rotary nanodevice.

**State**	**Large ring – ATTO647N**	**Small ring – ATTO550**
	**Distance to AuNP center (nm)**	
**‘I’**	12.5	16
**‘II’**	16	12.5
**‘III’**	24.5	16.5
**‘IV’**	32	22
**‘V’**	25	30
**‘VI’**	16.5	26
**‘VII’**	12.5	16

III. DNA Sequences

Supplementary Table S7. DNA sequences of the core staples for the small origami ring.

Core-1	TCCGGGAGAAGCCAATCAGAAAAGTAT
Core-2	GTCACGACGTTGTCCCCGGTCATAGCTGTT
Core-3	CTCATGGGTACCGAGCTCGAAAAATTCGCATT
Core-4	GGTCAATAGGGTTTGATAACCTAGCATTATGCCTGCAGGT
Core-5	TTATCCGCCCTTATAAATAGCGGTCGCCTGGCCCTGAGATTGC
Core-6	ACGTCATTTTTGCGGATCAACATGTTTTAAACA
Core-7	TAATTCGAGCTTCAAAGCG
Core-8	ACCGCGTTTATTATAGTCAGAAGCAAACGAGAATGATAT
Core-9	AAAAACCGTCTACATTCAACTAAGAGGAAGCAGGGCG
Core-10	TTGGAACAAGAGTCCACTATTAAAGA
Core-11	GCTTAATTGCCTTTACCCTGACT
Core-12	TCCGAAATCGGGCCCCAGGGCAACAGCTGATTGCTTTCTTTTCACCAGTG
Core-13	AGAGGTTGCAGCACAAAAGTTGTGTGAAATTG
Core-14	CTACGTTAATAAAACGAAGAGAATCGATGCTGAGAGTAGTAAAA
Core-15	GTATACTGCCCAGGGCGATCGGCCAGTGCCAAGCTTGCAACCTGCGCAA
Core-16	AAACTAGCATGTCACGTTGGGAAGTT
Core-17	TTGTATCAATAGGAACGCCGCCAGCTTACGTTGGT
Core-18	AATATTTAAATTATCAGCTCATTTGTGAGCGCGGATTGA
Core-19	TAACCGGAAGCATACAACTAACGTGATGGTGGT
Core-20	TTGTTATTCGTAAAATTCCATGAGCTAACTCACATTCTATTACGCCCA
Core-21	TTTTAGAACCCTCATATACTGAAAAG
Core-22	AAATTTATCGATTAAGTTGGGTAACGCCAGGGTTTTCCAGCTGG
Core-23	TCATCAATTCTACTAAAGGCAAGGGGCAAAGC
Core-24	GAGACAGTCAAATAACAGTTGATTC
Core-25	GTACAGGGTAGCTATTTTTGCGCATCGTGTCTGGCCT
Core-26	ATTAAGCAATAAAGCCTCAGAGCATAAAGCAGATCGCA
Core-27	TTAGCAAAGTGGCATTTGGGGCGCGAGTTTTAAATGCAATGC
Core-28	TATTACAGGTAGATCATA

Core-29	TCACCTCAGGATAAATCGGTTGTACCAAAGGATAAATGT
Core-30	CTGGAAGTTTCATTCCATATCACCATCAATATGACCATA
Core-31	TCCTGTAATCAAAAAAACAGGAAGA
Core-32	TCCTCGCAAATGGTCAACCATTAGATACATAATAGCCCGAGGGATTAG
Core-33	GTAGGTAAAGATTTTATTTCAACGCAAAACATTGACGACGA
Core-34	CGACTCTAGAGGATAAAACGACGGTGCGG
Core-35	GCTATACGAGTAGATTTAGTTGAGTGTTGTGCAA

Supplementary Table S8. DNA sequences of the fluorophore-modified DNA strands for the small origami ring. The fluorophores are attached to a dsDNA sequence (blue).

ATTO550-a	[ATTO550]ATGGAACGCAGGTCTCATCAACATTAAATTTTAACAAGCA
ATTO550-b	GAGACGAGGCATAGTAAGATGGATAGCCTATCAGGATGCGTTCCAT
ATTO647N-a	[ATTO647N]ATGGAACGCTCAGGATCCAATAAATCATACTAGTAGTGTTTA
ATTO647N-b	CCAACAACCTGTCGCATGCGTTCCAT

Supplementary Table S9. DNA sequences of the foothold staples for the small origami ring. The foothold region with AuNP-capturing sequence is highlighted in green. Footholds 1c and 4d have two functions, which additionally provide the double-stranded stem region for respective locking strands (red).

Foothold-1a	TCTGAATGGATTCGATCA CTCCAGCCAGCTTTCCGGCACCGCT
Foothold-1b	TCTGAATGGATTCGATCA AATCAAAATCAGGTTGAATATAAT
Foothold-2a	TCTGAATGGGTACTGTGT CAGTATCGGACCGTTCTAGCTGATCTTTAAAC
Foothold-2b	TCTGAATGGGTACTGTGTAGTTCAGAAAAGCGGAGACTTCAAATATGTGGACGGCCGCTGAGTAAAAT
Foothold-3a	TCTGAATGGTTAACAACCATCTGCCAGTGCCGGAGCCCGGTTGATTCAAAAGGGTGAGAAATCCAA

Foothold-3b	TCTGAATGGTTAACAACCTGAATCCCCTAATGCAGATACATAACGCCAAAACGTCATAAATATTCAT
Foothold-4a	TCTGAATGGAGTTGTTGAGTAGATGGGAGAGATCTACAAAGGGTCCAATA
Foothold-4b	TCTGAATGGAGTTGTTGACTGCGGAATGGAATTATTTAGGAATACCATCAGGAATCACCCCAAATAAT
Foothold-5a	TCTGAATGGAAATCCTGTCCGTAATGGGTCATTGCAACGGTAATCGTA
Foothold-5b	TCTGAATGGAAATCCTGTTGTTTAGACGCAACACTAAAGATTCATCAGAAAAAT
Foothold-6a	TCTGAATGGTCAAGTGTTAACGGAGTAACAACCCGTCGGTTGTTAAGTAAACGT
Foothold-6b	TCTGAATGGTCAAGTGTTACCCTCGTTTGGGGGTAATCTGGAGCAACA
Foothold-1c	TCTGAATGGATTCGATCAGTGCTGCAAGGTCTCCGTGGAAAAAGTGTAAAGCCTAAAGAAGTGACTACGAACTAGACCTAGTATCGATGACT
Foothold-1d	TCTGAATGGATTCGATCATAAAAACCAAAATAGACGCAGGCGAAAATCCTGTTGAACAACAT
Foothold-2c	TCTGAATGGGTACTGTGTCGAAAGGGGCTAATGAGCACAACATACGAGTATT
Foothold-2d	TCTGAATGGGTACTGTGTAGCGAGAGGCTTTTGCAGGGGTGCGAT
Foothold-3c	TCTGAATGGTTAACAACCGCCTCTTCGAATTGCGTTGCGCTCTGGGCGCC
Foothold-3d	TCTGAATGGTTAACAACCAGGGTGGTTCCTTCACCCACGCTGGTTTCAAAATCTCA
Foothold-4c	TCTGAATGGAGTTGTTGACTGTTGGGAGCTTTCCAGTCGGGAGCGCGGGG
Foothold-4d	TCTGAATGGAGTTGTTGAAGAGGCGGTGATACCTTTAATTGCTCCTTTTGCGGGACTACGAACTAGACCTAGTATCGATGACT
Foothold-5c	TCTGAATGGAAATCCTGTGCCATTCGCTGCCAGCTGTATGCAACTAAAGGGAAACCACAAAGAA
Foothold-5d	TCTGAATGGAAATCCTGTTTAATGAATATAAGAGTCCAACA
Foothold-6c	TCTGAATGGTCAAGTGTTTCTGGTGCCTACGGTGTCCAATTCTGCGAATTT
Foothold-6d	TCTGAATGGTCAAGTGTTGCTGTAGCTGGCTTAGAAACCAGACCGGAATCCAGT

Supplementary Table S10. DNA sequences of the locking strands for the small origami ring. The double-stranded stem region is colored red and the locking sequences are highlighted in orange.

Linker-1'a	AGGTGAGTAAGTTTTAGTCATCGATACTAGGTCTAGTTCGTAGTCGAG GGATGACCCTGTAATACTTTTGGCAACCGTGC
Linker-1'b	AGGTGAGTAAGTTTTAGTCATCGATACTAGGTCTAGTTCGTAGTCAAA TGAAATTAATTTGACTACGAACTAGACCTAGTATCGATGACT
Linker-2'a	CGTCAACCGAAATTGCATCAAAAAGATCTCGACTACGAACTAGACCT AGTATCGATGACT
Linker-2'b	GTACTTAGTGTCTTTAGTCATCGATACTAGGTCTAGTTCGTAGTCTTTG CCAGAACCAGACGACGA
Linker-3'a	TGTAGAGGATGATTTAGTCATCGATACTAGGTCTAGTTCGTAGTCTCA GGATCCAATAAATCATACTAGTAGTGTTTA
Linker-3'b	TGTAGAGGATGATTTAGTCATCGATACTAGGTCTAGTTCGTAGTCCCA ACAACCTGTCGCATGACTACGAACTAGACCTAGTATCGATGACT

Supplementary Table S11. DNA sequences of the core staples for the large origami ring.

Core-1	CGGCCAGAATCAGCCTGTGCACTCTGTAGCATCAGCGGGGTCAGACGAT
Core-2	CAGCACAGACGATTGGCCTTAACAGGAGGT
Core-3	TTGCTCGTCATAAACATCTGCATTA
Core-4	TCACCCTGCTGGTCTGGACCAGCAAGTTTCT
Core-5	CTCAATCAGGAATTATGAAGATAAAACACAGTGCCA
Core-6	CGGATTTGCGGGAGAAGCCTAATACCCTGTAATACATCTAAT
Core-7	TATACCCTTAGAATCCTTGAGACGCTATTA
Core-8	GCAACACCAAAAACATTATGATTAAAGCTA
Core-9	ATGCCAAATAATTCGCGTCTGACAATAGGA
Core-10	CGCTGAACAAAGAAACCACCATAACATTAT
Core-11	GTATAGGTAATCGTTTGATAAGCATGCAATGCCTGGTGAGA
Core-12	CATTTTGCAAATTAAATCCTTTGC
Core-13	CAGCATATCATCATATTCCGAAGAGAAGGAGCGGAGAAGAAG
Core-14	GCTGATAAACAACATGTTCTAAACCAGACGACGACTCAGGGC
Core-15	AATTTGAGGACTAAAGACTTCGAACGGCTA

Core-16	AAGGCCAGCTTTCATCAACACACGGCCTTCCTGTAACAGCAA
Core-17	CCGAACGTTATTAATTACTTTGAATAAAACAATTCGACAACTCGTCC
Core-18	CGGAACTGAGTTTCGTCACCGAAACCCATG
Core-19	AGAGCGGTTTACCAGCGCCAAATCAATAGA
Core-20	CAATATTTTGTTTAACGTCACACAATAAGAAACGAGGTTTTG
Core-21	CTTTAACTCAATCCGCCGGGATCATTGCAG
Core-22	ATCGAATTTATCCCAATCCAAAAAAATAAA
Core-23	CACCAAATAAATCCTCATTGTTAGATATTCACAAACTCCCTC
Core-24	GCATCAGGTGTGTTAGTTGAGCTACGTGAACCA
Core-25	GCGCGTTTTAAGAACTGGCGTACATTACCTTATGCTGAATCA
Core-26	ATAGTAAGATTAAGAGGAAGCTAAAGCGGA
Core-27	CGGGGCGCCTGTAGCATTCTGAGAGTACAAACTACCACGCCA
Core-28	GTAATACATTCAACCGATTTGGAAAGACAAAAGGGGGAAACG
Core-29	CGTAACCGGAATTTGTGAGTTATCATGTTTACCAGGGCAGGG
Core-30	AGTTAGTAAAGTAATTCTGTCGATAAAGTA
Core-31	CTTTCTTCCATTAAACGGGTGATTTTCATGAGGAATTAGGCT
Core-32	CACTCCGAAACGTACAGCGCCCCTCACCGG
Core-33	GCGACCTTTAATCATTGTGAATGAGATGGT
Core-34	GGCTGGAGGTGTCCGGGTCATACCGCGTCGGTGGTGCCATCCCACG
Core-35	ACATGTTAATTGCTTAGATTTCAATAACCTTCATTGAATCC
Core-36	AGGAGCCGGTGAATGTAACAGTTTCAGCCGAATAAT
Core-37	AAGGAATTCAACTACTTTTGCAATGTTTAGGGATATTCATT
Core-38	AGACTCCTCAGTACAATCAGTGCCTTGAGCCTATTT
Core-39	GTAGATGGTAATGGCGCCAGCGGA
Core-40	CTAGCTGAATCACCAGCAAACGCATGTCAAGTGGCATCAA
Core-41	GGAATTGGGAGTGACAGCTTGCCGATAGTTTCAGGAGGTTT
Core-42	GCGTCAGTAGCCCCCAGTAGCACCATTAATCGATAG
Core-43	GCCCCCTGTAACAGTAGCGGGCGAGAGGGTTCCCTCAGAGC
Core-44	CATTACCCTTATCCTTATCGGCTGTCTTCCGCACTC
Core-45	TGAAACCCCATTAGGGCATTTCTTTTCATATACGCAGTATG
Core-46	ACCGACCACCGGAAAATGCAAATCCAATTATATTTT

Core-47	ATTGTGTACGGAGACCAACTTGGCGCATAGATATATTCGGT
Core-48	GCCCTTTACCAGAACGTGAGCGCTAATACAATAATA
Core-49	CCAAGTATCCTTATTCAGATAGGCGTTTTACCAACGCTCA
Core-50	GAGGCATTTTCGAGCTTTAGCAAGCACCATATTTAACAACGCCAACAT
Core-51	TTTCAAACGCAAGAAAATAAGGCCTGTTTATAATTACATTT
Core-52	CGCCATATTAGCGTACCAGAGGGATCACCGTCACGCAAAAT
Core-53	TTAACGTATAAAGAAAGTTACTTACCTGAGATACATTTGAG
Core-54	AGTACGGCGGATAACCGGAATCTCATACATGGCTGTTTTAA
Core-55	TGAGTGAATAACCTTCTAATAAGGCGTTTTAATGGAAACAGTACAAC
Core-56	TTAGCAAACCGAGGCTGGCATGGGCGCATTAGACTCAGAGG
Core-57	TTCTAACATTTCGCCATTTGGGCGATTAGAGAGTATTTTTG
Core-58	ACCCAAACGGTGTATCAAGAGGTAAAGAATACACATACCAA
Core-59	GCGCTTTCGCCGCAACCAGCTTAC
Core-60	AAACAATCGAAGTGCCAAGCTTTC
Core-61	ACGCCATCAGAAATTCGCATTAAA
Core-62	AATCGGTTTACAAGGCAAAGAATT
Core-63	TTGCATCAGAAAATCAAAAATCAG
Core-64	TTAATTTCAGCTTGCCCTGACGAG
Core-65	CAGAGGCTCAGTCACCCTCAGCAG
Core-66	TACCGTAAGCTCAGAGCCACCACC
Core-67	TGAGGCAGTTAGCCGCCACCAGAA
Core-68	AAATTCATCGAAAGAAACGCAAAG
Core-69	CAGCCATAGAAACGCTAACGAGCG
Core-70	CCGACAAAAAATGTAATTTAGGCA
Core-71	ATTAATTTTATAAATCAATATATG
Core-72	AGCCGCACATCGACGTCTCGTTAACGGAACTTAAGTTGGGT
Core-73	GCCTCAGCAGTTTGGGCTGCGCTTCGCTATACAGGAAGATT
Core-74	TTGCTGGTAATATCCAGAA
Core-75	AGAGCCACGACTTGCAAAATCAGG
Core-76	TGCTATATTCTAAGTGCGGGATAAGAAAAATAATAACCAAT
Core-77	AGAGGTGGAGCCGCCGTTCCGTTTTTGACGTTGTAAAACGACGGCCAA

Core-78	TATCAGGTCGATGAGCCGGAGACAGTCAATAAATTA
Core-79	TTTTTGTTAAATCACATTCGCCATTAAACGTTAATATTTTGTTAGC
Core-80	TCTACAAAAGGAGAGGGTAGCTATTAAAGCCTCAGAGCATCAACCGTT
Core-81	AGCAAAATTAAGCAATTTGAGAGTCTCATCCAATAAATCATACAGGAC
Core-82	ACGATAAGTTTTGCCAGGAATACCACATTACGAGGC
Core-83	GTCTTTACCCTGACTGGCTGCGAACGAGAAAACGAGAATGACCATCG
Core-84	GGTCAATGGACAGAGAAAACAAAGTACACGAAATCC
Core-85	AAACACCAGAACGAGATATAGCGAGAGCTCATTCAGTGAATAAGGAC
Core-86	TAATAACATCACTTGAAAACAAAATGTTTAGGTAAAATCATAGGT
Core-87	CGAAAGACAGCATCTAAACCGAACTAGGCCGCTTTTGCGGGATCAA
Core-88	AAGGCTCTTTTTCACGTTGAAAATAGCAAGCCCAATAGGACAACTAAA
Core-89	CTCATTTTCAGGGATCTTATCGGTTTCACCCTCAGAACCGCCACCCTG
Core-90	GATTTGGAAGGTTAATAGATTTGGAACTGATAGCGAACCAC
Core-91	CCACCACCAGAGCCCAAGGATTAGGTCAGAGCCACCACCCTCAGTA
Core-92	TATTAGACTTTACCCAATTGCGTACCTAC
Core-93	ACACCACGGAATAAGACGCGTTTTCACATAAAGGTGGCAACATATAAA
Core-94	ATCTTACATAAGAAACAATGAAATAATTTGCCAGTTACGAATTGAG
Core-95	TCTTTCCAGAGCCTAGAGTAAGCAGTTTTATCCTGAATCTTACCAT
Core-96	ATCGTAGTTGAACAAGCAAGCCGTCAGTAATAAGAGAATGGTATTAAA
Core-97	CGCTGCTTAAACAGACAACCAATTCCAGACGTTATAAACAA
Core-98	CACCCAGCTACAAATGATAACCCACCCTG
Core-99	TAGAAAATACATATCCAAGGCCGGGAATT
Core-100	TTAATTGAGAATCGAACATTCCAAGCGAGC
Core-101	CCCTCAGAACCGCATGAATAGAATATGGG
Core-102	TGCAGGGAGTTAAGATTTGTATCACCCCC
Core-103	AGAACCGCCACCCATTGCCCGTAGTACTG
Core-104	GTCCATCCGACTAACGCGATAGAAGGGCGCCCAATTATTTTC
Core-105	ACAGTATAATTACTTACAAATAAACCTTTTTAACTGTAAAT
Core-106	CTTTAAACAGTTCAGGAATATAATTGATA
Core-107	AGTAGTAGCATTAAGGATCAATATGAAGAT
Core-108	AGTTGATCCATTAGTTGGCTTAGAGCTTTTAAATAT

Core-109	ACGTAACAAAGCTGGATGCAGATAGAAAG
Core-110	ATATTTAAATTGTCAAGGGGACGGTTGGT
Core-111	TTTTCCCAGTCACTCATAAAAAATCTGCT
Core-112	TTTGAATTACCTTTTCAAAGAACTTATAT
Core-113	GGGGAAAGCCGGCCCCCGGAAAACTAAAGAGAATCATTGCCTTGAGAG A
Core-114	TTGCTTTGACGAGATGACACTTGATACTTTCGATTTAATTGCCAAAAAA
Core-115	CCAACGGCAGCACGGTGCCCCCTTTAACG
Core-116	TAAAGTTTTGTCGTCTTGCCACTAC
Core-117	CATTTGCGGATCAAGCCGGACCGAGGTGCCGT
Core-118	CCAGCGCGTGCCGGGGTTTCTAGCCTCCTCTCAGCAGCAAC
Core-119	TGGGAAAGTCATTATACCATAATCTCACCCG
Core-120	CATATCAATAATGGAAAGAAGAACTATCGGCC
Core-121	AACTATACTCCGGCATCATATTAG
Core-122	GTAATAATTTGATGATATAAGGGA
Core-123	ATTTTGCGTAAATGGCCGACACACGTATAACG
Core-124	AACAAAGGGGAGAAATACCCATAATCAGTGAGG
Core-125	ATGTAGAATCCCATGAACCTCCACGCAAATTA
Core-126	TCAAAAGGAGTAATATATGTAGAACGTGGCGA
Core-127	CTGAGAGACTCGCGCCTGT
Core-128	CCAGGCATGGGAAGTCCCGTGCATCTGCGAAGATCG
Core-129	ATTCATCACAACATTGGATAGGCT
Core-130	TTCCGGCAGCCTCCATGGCGGTTGTGTACAGGCGGC
Core-131	TTAAGCCTCAGAGAAGCCGAAATAATAACGTCAAGATTAGT
Core-132	GGATTCGCGCAGAGAGCGTAAAACAGAACAGATGAA
Core-133	AACGCCGATCGGTGAAGGGGGCTGCGGATTGACCGGCGCAT
Core-134	TCCACAGGCGGGCCGCGCGTGCCTCTGCAGCCAGCGAGTGTCAC
Core-135	CCGGGCAGCCAGCTTTCCGGGCTCATTTTTTAACCAGTATCG
Core-136	GTATTAAGAGCACCTATTATTCTGAAAGCCGCCAGCATTGACAGTTAAT
Core-137	AGCGATTTAAAACATGGCTGAGCC
Core-138	GCCGTCCGTAATCAGTAGCGTTTATTTTGTCACAACGTCACCAA
Core-139	ATTGCGTGATGAAGGGTAAAACGGGAACGGATAACCGTAAAAAA

Core-140	TTTTATTTGCCCGAAAGACTTCAAAATG
Core-141	GGGGTGCCTGAGAGAGTTGCAGCAAGCGGTCCACGCTGTCTGGCCAAC A
Core-142	CCAAAAAGAGCAACACTATCTAGTAAATTGGGCTTAACGCCAA
Core-143	AGACGGGGCAAACATAGCGATGCTTTCA
Core-144	GGAGAAACAATCAGTAACAGTACCTTTTTAAAAGTTTGAGTTTTCAGG T
Core-145	TGCGCCATTAATTGCGTTGCCTAATGAGTG
Core-146	CAGCAGGAAGCCTAGTGTACGAAATCCCTTTATCCGCTCACAAT
Core-147	AGCTAACTGCCAACATACGAGCCGGAAGCATAA
Core-148	CACTGCGGCCGCGGTTGCGGTGGGAATG
Core-149	TAATGGTTTGAGATTTCATCTTCTGACGCTTCTGTAAATCGTGAAAACTT
Core-150	GGAAAGTCCGCTCACTGCCCGCTTTTCCTGTGTGAAA
Core-151	TCATTCCATAGTTAAAGTACGGTGTCTATTATAGTCAGAAGCGTAGCTC A
Core-152	TCAGGAGAGCCAGCAGCAAAT
Core-153	TCCTGAACAGCAGCCTTT
Core-154	GTCAATAGATACAAAGGGTATATAATCCTGA
Core-155	TTGTATCCGCCTGGTTTTCTTTTCACCAGTGAGACGGGCCCTGAAAGCGT
Core-156	AACAAGAATTATTCAAACAAACATATACTGGA
Core-157	AAGGGAAAAGATAGACTTTATGTGCCTAAATCGGAACC
Core-158	CCCTCGAGGGGGTAATACTGCAAGAACTAAAAC
Core-159	CGCCTGATACAGGAATTTACCGTTCCAGTAAGCG
Core-160	CGCAACCAGAGCACAACGTCACTGCAGAGAGA
Core-161	GCTAAACAGGAGGCCCCGGAAC
Core-162	AAAGCATGCAAGGCGAGTACTTATAGCTCTCACGG
Core-163	GCGCGTAACCACCATGACAAGAACCACTATTACGAAAAATCTAC
Core-164	TCTTCGCCGGGTACAGCCCTTACACTGATGCCGGGTTACTTCCAGTCG
Core-165	ACACGAGCATCATCTAAACTAACACCGCCTGCAA
Core-166	GAGAATCAGTAATAAAAGGGACATGTTTGCCC
Core-167	GCCCGAGATCGCTCAATCGTCTGAAATCAGGCGGTCAAAAAAT
Core-168	TCACCTACATTTTGAAGGGTTGAG

Core-169	AACGTCCCACTATTAAAGAACAAAAACGC
Core-170	GAAAAACCTTGCTGTCAGTTGGGGATTATTTACATTGGCAGTGGTTCCG A
Core-171	TGTTGTTAAGAATAAATCGGCAAAAATCCTGTTTGATGGATTCACCAGT C
Core-172	TACGTGGCACAGGTTTGCGTATTGGGCGCCAAGAGTAAAGGGCGAAA
Core-173	ATTGCCCTTCAAAATCAACCAGTTTGGATCATGGTCATAGCTGGT
Core-174	AAGTTGTTAGAACCCTTCTGAAACAGCTG
Core-175	GGGAGAGGCGACAATATTTTTGACAAA
Core-176	GAGGCGCAACTGCTCCATGTTACTGGAACGAGGGTAGCCCTGATAA
Core-177	CCGCGCTTAATGCCCTTCACAGACCATGAAAGACATAAGGGGCCGGAA C

Supplementary Table S12. DNA sequences of the fluorophore-modified DNA strands for the large origami ring. The fluorophores are attached to a dsDNA sequence (blue). The complementary sequence to hybridize the ATTO550-a strand is provided by Foothold-4d.v2 (see Supplementary Table S13).

ATTO647N-a	[ATTO647N]ATGGAACGCAGAGTAACAACCCGTTCATTAAATGTGAT CAGAACTTGAC
ATTO647N-b	ACCCTCATATATTTTAGCGTTCCAT
ATTO550-a	[ATTO550]ATGGAACGCGACGGATTGAGGGAGGGAAGATTAAATCCT GAGAAGTG

Supplementary Table S13. DNA sequences of the foothold strands for the large origami ring. The foothold region with the AuNP-capturing sequence is highlighted in green. Footholds 3, 4 and 5d, e and f have a two-nucleotide thymine extension (dark orange) placed between the foothold sequence and the DNA origami structure. Foothold 4d has several functions. It serves as a linker (red: stem section of the locking strand; orange: locking sequence; purple: toehold sequence) and provides the hybridization sequence for the ATTO550 dye (Foothold-4d.v2, blue), respectively.

According to whether only the large origami ring or the entire rotary device is assembled, the addition of foothold strands has to be adjusted.

Foothold-6a	TCTGAATGGTCAAGTGTTATGAATTGCCTGTCGTGCCCGAGCTCTA
Foothold-6b	TCTGAATGGTCAAGTGTTAACCGTCGAATTCGTAAACAGGGCGGCCA ACGCGCG
Foothold-6c	TCTGAATGGTCAAGTGTTTCAGGGCGATGGCCCAGATCCCGTCCGTG GCCAGCATTTTCACGTGCTG
Foothold-5a	TCTGAATGGAAATCCTGTCGGTAATGGGTAAAGATTTGCCCTGCGGG CGTGGAAATCAAGTTTTT
Foothold-5b	TCTGAATGGAAATCCTGTAAAAAAACAGCCGCCAGCAGTTGGAGCC GGGT
Foothold-5c	TCTGAATGGAAATCCTGTTGGGGTTTGTAGATCCTCACGCTGGCAAA CGCGGTAAACGATGCTG
Foothold-4a	TCTGAATGGAGTTGTTGAATTCTCCGTGGGAACAAACGCCGTGGTG
Foothold-4b	TCTGAATGGAGTTGTTGACTAAAGTGGCGACGGGCCTCAACTGTAAG CGCCACCGCTTCTGGTG
Foothold-4c	TCTGAATGGAGTTGTTGAGCCCCCGATTTAGAGAAGCCCCAAAATAG ATAGGCGG
Foothold-3a	TCTGAATGGTTAACAACCAAATTTGGTTTATTTCAACGGCGCGAAG
Foothold-3b	TCTGAATGGTTAACAACCGAAAGGAGCTGAAAAGTCGTGTATTAGA
Foothold-3c	TCTGAATGGTTAACAACCGGAAGAAAGCGAAAGGTATTTTAAATGG TAGTTTGATCCCAATTAAGTT
Foothold-2a	TCTGAATGGGTACTGTGTAGCGAACCAGACCGCCATTCGAGCTTCGG AATCTGGCAAGTGTAGC
Foothold-2b	TCTGAATGGGTACTGTGTGTTAATAACGGAAGTTGAGATTTAAATAT CGCG
Foothold-2c	TCTGAATGGGTACTGTGTGGTCACCGTCCAATAGTAAAAAAGAAAA ACCAAAAACCCTCGTTTA
Foothold-1a	TCTGAATGGATTCGATCAAACCTAAAACGAAAGAGGCACAGGACGT
Foothold-1b	TCTGAATGGATTCGATCAGAAGGCTTTAAAATACGTATCGCCCCTAT GG
Foothold-1c	TCTGAATGGATTCGATCAGCTACAGGGCGCGTAACGCATAACCGGC CTCATCACC
Foothold-6d	TCTGAATGGTCAAGTGTTTCATAGTTAGCGTAATCCACAGACAGCCA GGTGTCCT

Foothold-6e	TCTGAATGGTCAAGTGTTTGCTTTATCACCGTACGCAATTTCGATC
Foothold-6f	TCTGAATGGTCAAGTGTTCGTTAGAATCAGAGCGTATAGCGTGCCGTGTTTTGCTCAAGAGATGAAA
Foothold-5d	TCTGAATGGAAATCCTGT**TT**AAGGTGAATTAAAGCGCAGTCTCTGAAAAGCCAGAATCCACCAGATTAA
Foothold-5e	TCTGAATGGAAATCCTGT**TT**TTAGACACCGGATTGCCATTCGGTCAACTGTAGCAGAATCAAGTTT
Foothold-5f	TCTGAATGGAAATCCTGT**TT**AACGGTACGCCAGAGACTCCTTATATAGCCAAATTATTCATTA
Foothold-4d	TCTGAATGGAGTTGTTGA**TT**AATAACATAAAAACAGGGAATAAATATTCTGATGCTTGATCTGGATCATAGCTACTGATTTACTTACTCACCTAAACACACATCA
Foothold-4d.v2	TCTGAATGGAGTTGTTGA**TT**AATAACATAAAAACAGGGAATAAATATTGCGTTCCAT
Foothold-4e	TCTGAATGGAGTTGTTGA**TT**ACAGAGAAAAAATGAAAAGGTTTTAGTAAAAGAGTCT
Foothold-4f	TCTGAATGGAGTTGTTGA**TT**CCACCGGAAGCCTTAAAGATTAACTGAG
Foothold-3d	TCTGAATGGTTAACAACC**TT**TTATCTTAGCTAATGCAGTCTTACGTA
Foothold-3e	TCTGAATGGTTAACAAC**TT**ACCGTTCAGTATAAAGGCCCTAAAACAATAGATAAG
Foothold-3f	TCTGAATGGTTAACAACC**TT**GCAATACTTCTTTGATGCGTTAAGAAAAAAATAAACGTGTGATAAAATT
Foothold-2d	TCTGAATGGGTACTGTGTTATAGTGAATTTATCTGCTGAGAAGAGTCATCAAGCC
Foothold-2e	TCTGAATGGGTACTGTGTTTGTTTTCTGAAAATTATTTGCACTTAGATTA
Foothold-2f	TCTGAATGGGTACTGTGTTGAGTAGAAGAACTCAATGATGATTTCAAAAAATCGCCTGATTGCATCG
Foothold-1d	TCTGAATGGATTCGATCATTTAATGCGCATGGCAATTCATCATATGATTATCAGAAGAGCC
Foothold-1e	TCTGAATGGATTCGATCACAATATCAACTATCTAAAAAGTTGAAAATATCTGGAACCTCAAATA
Foothold-1f	TCTGAATGGATTCGATCACGCCAGCCATTGCAACAGGGTGGACTCC

Supplementary Table S14. DNA sequences of the locking strands for the large origami ring. The double-stranded stem region is colored red and the locking sequences are highlighted in orange. Toehold regions to initialize the toehold-mediated strand displacement reactions to open the locks are colored in purple. Foothold strand 4d (see Supplementary Table S13) serves as an additional lock.

Linker-3a	TCAGTAGCTATGATCCAGATCAAGCATCAGAACAGGTCAGAAGGATA A
Linker-3b	TGATCCAGATCAAGCATCAGGGCGCTAGGGCGCGTCATAAATATGTA TTGCTGAAGCAAACTCCCTGATGCTTGATCTGGATCATAGCTACTGAT TTTCATCCTCTACAACTATCTACTC
Linker-3c	AGAGGTCACCTTTATTAGCTAAGCGCTGATGCTTGATCTGGATCATAG CTACTGATTTTCATCCTCTACAACTATCTACTC
Linker-1a	AGCTATGATCCAGATCAAGCATCAGGACGGATTGAGGGAGGGAAGAT TAAATCCTGAGAAGTGCTGATGCTTGATCTGGATCATAGCTACTGATT TACTTACTCACCTAAACACACATCA
Linker-1b	TCAGTAGCTATGATCCAGATCAAGCATCAGTTTTTAAAAGAAAAACG CACAAAGTTTTAAGAAACAATAGCT
Linker-2a	TCAGTAGCTATGATCCAGATCAAGCATCAGGCTATTAGTC
Linker-2b	GAGGTGAAATCAACTATCTTTAGAATATGGAGACATCGCATGCTGAT GCTTGATCTGGATCATAGCTACTGATTTGACACTAACTCAAGTGTTCG AGTA
Linker-2c	ACCGAACCCTAAAAGCACTAATACCTGATGCTTGATCTGGATCATAGC TACTGATTTGACACTAACTCAAGTGTTCGAGTA

Supplementary Table S15. Unlocking strands to open the locks of locking couples L1-L1', L2-L2' and L3-L3'. Toehold regions are colored in purple.

Open-L1	TGATGTGTGTTTAGGTGAGTAAGT
Open-L2	TACTCGAACACTTGAGTTAGTGTC
Open-L3	GAGTAGATAGTTGTAGAGGATGA

Supplementary Table S16. Foot strand for the surface modification of the AuNPs. The capture sequence to bind to the footholds is colored in green. The thiol-modification is shown in brackets.

Foot	CCATTCAGTTTT[ThiC3]

Supplementary Table S17. DNA sequences of the blocking and releasing strands. Blocking strands: green, foothold binding sequence; purple, toehold region. Releasing strands: purple, complementary toehold; green: sequence to deactivate blocking strands.

Blocking-1	TGATCGAATCCATTCCTTCACTTTCA
Blocking-2	ACACAGTACCCATTCCTACCTATGT
Blocking-3	GGTTGTTAACCATTCAATGTACTTAC
Blocking-4	TCAACAACTCCATTCATAGCCTGTGA
Blocking-5	ACAGGATTTCCATTCCCGATTATTAG
Blocking-6	AACACTTGACCATTCCTAACTTCTTA
Releasing-1	TGAAAGTGAAGGAATGGATTCGATCA
Releasing-2	ACATAGGTAGGAATGGGTACTGTGT
Releasing-3	GTAAGTACATTGAATGGTTAACAACC
Releasing-4	TCACAGGCTATGAATGGAGTTGTTGA
Releasing-5	CTAATAATCGGGAATGGAAATCCTGT
Releasing-6	TAAGAAGTTAGGAATGGTCAAGTGTT

IV. References

1. Douglas, S. M.; Marblestone, A. H.; Teerapittayanon, S.; Vazquez, A.; Church, G. M.; Shih, W. M., Rapid Prototyping of 3D DNA-Origami Shapes with Cadnano. *Nucleic Acids Res.* **2009,** *37* (15), 5001-6.
